# Supplementary material for: Phosphorylation of POU3F3 Mediated Nuclear Translocation Promotes Proliferation in Non‐Small Cell Lung Cancer through Accelerating ATP5PF Transcription and ATP Production
Source: Adv Sci (Weinh). 2025 Feb 11;12(13):2411503. doi: 10.1002/advs.202411503 (PMC11967767; doi:10.1002/advs.202411503)
Supplement: Supplementary file 1 — Supporting Information [file ADVS-12-2411503-s002.docx]

**Phosphorylation of POU3F3 mediated nuclear translocation promotes proliferation in non-small cell lung cancer through accelerating ATP5PF transcription and ATP production.**

†Qi-Gang Zeng^1^, †Le Li^1,2,3^, †Tao Chang^1,2,3^, †Yong Sun^1,2,3^, †Bin Zheng^1,2,3^, Ling-Na Xue^1^, Chao-Ling Liu^1^, Xia-Qing Li^4,5^, Ruo-Tong Huang^6^, Jia-Xin Gu^2^, Zhao-Rong An^2^, Hao-Tao Yao^2^, Dan-Yang Zhou^7*^, Jun Fan^1,2,3*^, Yong Dai^1*^.

^1^ Nanhai hospital of Traditional Chinese Medicine, Jinan University, Guangdong, China.

^2^ Department of Medical Biochemistry and Molecular Biology, School of Medicine, Jinan University, Guangdong, China.

^3^ Key Laboratory of Viral Pathogenesis & Infection Prevention and Control (Jinan University), Ministry of Education, Guangdong, China.

^4^ Institute of Nephrology and Blood Purification, The First Affiliated Hospital, Jinan University, Guangdong, China.

^5^ Nephrology department, The Fifth Affiliated Hospital (Heyuan Shenhe People’s Hospital), Jinan University, Guangdong, China.

^6^ Department of Metabolism, Digestion, and Reproduction, Faculty of Medicine, Imperial College London, London, UK.

^7^ Department of Respiratory, Nanjing First Hospital, China Pharmaceutical University, Jiangsu, China.

† These authors contributed equally to this study.

^*^ Corresponding to:

Yong Dai, Ph.D

Nanhai hospital of Traditional Chinese Medicine, Jinan University, Guangdong, China.

Tel: 13927780632;

E-mail: daiyong249@163.com

And

Jun Fan, Ph.D

Department of Medical Biochemistry and Molecular Biology, School of Medicine, Jinan University, Guangdong, China.

Tel: 13533785802;

E-mail: fanjun@jnu.edu.cn;

And

Danyang Zhou, Ph.D

Department of Respiratory, Nanjing First Hospital, China Pharmaceutical University, Jiangsu, China.

Tel: 15380998936

E-mail: danyangzhou1986@163.com

**Abstract**

Targeting oxidative phosphorylation (OXPHOS) through inhibiting the electron transport chain (ETC) has shown promising pre-clinical efficacy in cancer therapy[1]. Although aerobic glycolysis is a hallmark of cancer, emerging evidence suggest OXPHOS is frequently enhanced, even in the presence of active glycolysis, providing metabolic advantages for cell proliferation, metastasis and drug resistance in a variety of aggressive cancer types including non-small cell lung cancer (NSCLC), yet the underlying molecular mechanisms remain elusive[2]. Here we report that POU-domain containing family protein (POU3F3) is translocated into the nuclei of NSCLC cell lines harboring mutant RAS, where it activates transcription of ATP5PF, an essential component of mitochondrial ATP synthase and consequent ATP production, leading to enhanced NSCLC proliferation and migration. Moreover, we further found out that ERK1 phosphorylates POU3F3 at the S393 site in the cytoplasm and promotes the nuclear translocation of POU3F3 via receptor importin β1 in RAS mutant NSCLC cells. Mechanistically, RNA sequencing analysis combined with chromatin immunoprecipitation (ChIP) assay revealed that POU3F3 binds to the promoter of ATP5PF, leading to enhanced ATP5PF transcription and ATP production. Together, our study uncovers a novel RAS-POU3F3-ATP5PF axis in facilitating NSCLC progression, providing a new perspective on the understanding of molecular mechanisms for NSCLC progression.

**Key words:** NSCLC; POU3F3; ATP5PF; OXPHOS

**Introduction**

Lung cancer is the most commonly diagnosed cancer and the leading cause of mortality from cancers across the world[3]. Lung cancers are divided into two broad categories according its molecular characteristics, including small cell lung cancer which contributes 11% of incidences, and NSCLC which is further subcategorized into squamous cell carcinoma (LUSC), large cell carcinoma (LCC) and adenocarcinoma (LUAD), comprising 89% of all lung cancer cases[4]. As the most prevalent type of lung cancer, NSCLC was characterized by high morbidity and mortality. Although treatment for NSCLC has advanced significantly with personalized approaches including targeted therapy and immunotherapy, the 5-year survival rates of NSCLC patients remain poor[5]. This suggests that the underlying molecular pathological mechanisms of NSCLC initiation and progression are largely unclear. Focusing on understanding NSCLC biology and identifying an alternative target will be key to enhancing treatment outcomes for NSCLC patients.

Otto Warburg proposed that cancer cells prefer glycolysis even in oxygen-rich environments due to impaired mitochondria function, leading to reduced OXPHOS activity. However, this concept has been challenged for decades, as several cancer cell types have exhibited heightened OXPHOS activity, accompanied by comparable levels of TCA cycle intermediates and ATP to non-transformed cells[6]. Although increasing evidence has indicated the reprogramming of cancer cell metabolome to support cellular proliferation[7], recent findings indicated the coordinated crosstalk between glycolysis and OXPHOS collectively promotes the advancement of colorectal cancer[8]. Moreover, previous study shows that targeting OXPHOS and the electron chain conduce to inhibit lung cancer progression[9]. Hence, it is of great significance to investigate the aberrant activity of the OXPHOS and its underlying molecular mechanisms in NSCLC progression.

One of the most prevalent oncogenes in human cancers is mutant RAS, which is implicated in around 19% of tumors[10]. Mutant RAS proteins activate downstream effectors and play a pivotal role in driving oncogenesis. Cancer cells harboring mutant RAS exhibit aggressive phenotypes[11], wherein activation of the RAS-RAF-MEK-ERK cascade leads to uncontrolled cell proliferation[12]. Phosphorylation of ERK and translocation to nucleus is essential for cancer formation by enhancing oncogenic signals in many cancer types. However, whether nuclear localization of ERK promotes cancer cell proliferation by regulating mitochondrial OXPHOS is not fully understood[13]. The inhibitors targeting the RAS-RAF-MEK-ERK pathway deserve more attention in the current cancer research and treatment. However, drug resistance eventually develops at least in part due to additional activation of the bypass signaling and downstream effector molecules. For example, the nuclear translocation of ERK1 and ERK2 phosphorylated the Mitogen- and Stress-activated Protein Kinases (MSKs). Subsequently, MSKs phosphorylate and activate Activator Protein component (ATF1) at Ser63, initiating gene transcription[14]. These contributed to the pathogenesis of NSCLC[15]. Understanding and addressing the underlying mechanisms is crucial for developing strategies to overcome resistance and improving NSCLC treatment outcomes[12, 15].

POU3F3 encodes a POU3-domain containing protein that functions as a transcription factor, which recognizes octamer sequence in the genome. The expression of POU3F3 is highly and widely observed in the central nervous system, where it plays a crucial role in neuronal development through its collaboration with SOX4[16]. Remarkably, the differentiation of lung cancer into a proneural/neuroendocrine phenotype has been observed in approximately one-third of cases and likely exert a significant impact on tumor progression of lung cancer. It has been reported that POU3F2 might be involved in aggressiveness of lung cancer[17]. It suggests the possibility that other class Ⅲ/Ⅳ POU genes may function as a key factor to promote the lung cancer progression. However, the precise role of POU3F3 in the pathogenesis of NSCLC and its underlying molecular mechanisms remain elusive.

In this study, we found that endogenous POU3F3 is upregulated in NSCLC tissues and cells, and high expression of POU3F3 correlated with poor survival prognosis. Our xenograft experiment data show that mice injected with POU3F3 knockout H1299 cells exhibited a reduction in both tumor volume and weight, accompanied by a significant downregulation of Ki-67 in the tumor tissue sample, indicating POU3F3 promotes cell proliferation and tumor growth in NSCLC. Mechanically, KRAS^G12D^ enhances the interaction between ERK1 and POU3F3, thereby facilitating the phosphorylation of POU3F3 at S393 site and promoting its translocation to the nucleus through importin β1. The nuclear-localized POU3F3 binds to and enhances the transcription of ATP5PF, a component of ATP synthase, thereby upregulating ATP synthase activity and cellular ATP levels in NSCLC. This, in turn, promotes the proliferation of NSCLC cells. Overall, our research reveals an unidentified role of POU3F3 in NSCLC by regulating OXPHOS, which provides a novel insight of NSCLC progression.

**Materials and Method**

**Cell culture, plasmid construction and transfection**

All cell lines were purchased from American Tissue Type Collection (ATCC) and tested at least once for mycoplasma. The H460, H1299, H522, PC9 and H1975 cells were cultured in RPMI-1640 medium (GIBCO, Carlsbad, CA USA) supplemented with 10% fetal bovine serum (FBS, ExCell Bio, FND500, China), and maintained at 37℃ in a humidified atmosphere containing 5% CO2. The culture medium also contained 100U/mL penicillin and 100U/mL streptomycin. The HEK293, A549 and Beas-2b cells were cultured in Modified Eagle medium (DMEM, GIBCO, Carlsbad, CA USA) supplemented with 10% FBS, and maintained at 37℃ in a humidified atmosphere containing 5% CO2. The culture medium also contained 100U/mL penicillin and 100U/mL streptomycin.

CRISPR-cas9 was used to knock out POU3F3 in the human H1299 and A549 cell lines. A pairs of single guide RNA (sgRNA) sequences for human POU3F3 and importin β1 were designed by using the design tool from Feng Zhang Lab, then, the sgRNA was ligated into the LentiCRISPRv2 vector. The sequence of sgRNA was listed in Table S1. Exogenous human wild type (WT) and point mutation POU3F3 CDS sequence were cloned into pLVX3 plasmid for Flag-tag at C-terminus. Exogenous human ATP5PF CDS sequence was cloned into pLVX3 plasmid for Flag-tag at N-terminus. Exogenous human ERK1 CDS sequence was cloned into pLVX2 plasmid for 6×HA-tag at C-terminus. For luciferase assay, exogenous human ATP5PF promoter and corresponding mutation sequence were cloned into pGL3-basic plasmid. All mutants were generated using overlap extension Polymerase Chain Reaction (PCR). In brief, utilize PCR with pairs of oligonucleotide primers that incorporate the intended mutation. Subsequently, the PCR product was purified and extended, followed by a second round of PCR amplification to yield the full-length sequence containing the mutation.

For lentiviral infection, the HEK293 cells were seeded onto plates and cultured for 12-24 hours using DMEM containing 10% FBS until reaching a confluence of 60-80%. To generate lentivirus particles, HEK293 cells were transfected with the target plasmid (3μg), along with pSPAX2 (1.5μg) and pMD2.G (0.5μg), using Lipofectamine 2000 transfection reagent (Invitrogen, USA) following the manufacturer's protocol. Puromycin was added 24 hours post-transfection, and stable cells were cultured in a 1640/DMEM medium supplemented with 10% FBS. The efficacy of knockout was confirmed by Western Blot analysis.

**The RNA extraction and subsequent real-time PCR analysis is employed to quantify gene expression levels**

Total RNA was extracted from Beas-2b, H1299 and A549 cells using Trizol (Invitrogen, 15596018, USA) following the manufacturer's protocol, and the concentration was determined using a Nanodrop spectrophotometer (Thermo Fisher, USA). The extracted RNA (500ng) was reverse transcribed into complementary DNA (cDNA) using PrimeScript RT reagent Kit (TAKARA, RR037A, Japan), following the manufacturer's protocol. The quantitative real-time PCR (qRT-PCR) analysis was conducted using the LightCycle 480 SYBR Green 2 Master kit (Roche) on a CFX 96 Real-Time system (Bio-Rad). GAPDH expression levels were assessed as an internal control and utilized for data normalization. Primer’s sequence was listed in Table S2. RNA expression was normalized against the relative value from the negative control (NC) group. All experiments were replicated at least three times with n=3 samples per experiment.

**Western blot**

The cells were washed twice with ice-cold PBS supplemented with PMSF, followed by lysis on ice in RIPA buffer containing 1×protease inhibitor cocktail and 1×phosphatase inhibitor (Roche, Germany) for a duration of 30 minutes. The concentration of total protein was determined using a BCA protein assay kit. Whole cell extracts were boiled at 100℃ for 10 minutes in loading buffer containing dithiothreitol (DTT), and resolved by standard methodology on a 10% SDS-polyacrylamide gel electrophoresis system, followed by transfer to a PVDF membrane (Millipore). Blots were incubated with antibodies against POU3F3 (Proteintech, 18999-1-AP, 1:1000 dilution), ERK1/2 (Proteintech, 11257-1-AP, 1:1000 dilution), phospho-ERK1/2 at Thr202/Tyr204 (p-ERK1/2, Proteintech, 28733-1-AP , 1:500 dilution), MEK1/2 (Proteintech, 11049-1-AP, 1:1000 dilution), phospho-MEK1/2 at Ser217/221 (p-MEK1/2, Cell Signaling Technology, #9154, 1:1000 dilution), ATP5PF (Proteintech, 14114-1-AP, 1:1000 dilution), ATP5PB (Proteintech, 15999-1-AP, 1:1000 dilution), ATP5PD (Proteintech, 17589-1-AP, 1:1000 dilution), Lamin B1 (Proteintech, 12987-1-AP, 1:1000 dilution), HA-tag (Proteintech, 51064-2-AP, 1:3000 dilution), Flag-tag (Proteintech, 20543-1-AP , 1:3000 dilution), Phosphos-serine (Abcam, ab308512, 1:500 dilution), β-actin (Proteintech, 66009-1-Ig , 1:1000 dilution), GAPDH (Proteintech, 10494-1-AP, 1:1000 dilution), Signals were detected, with luminol reagent, using a HRP Substrate Luminol Reagent (Millipore).

For protein immunoprecipitation (IP), cells were washed twice with ice-cold PBS supplemented with PMSF, followed by lysis on ice in RIPA buffer containing 1× protease inhibitor cocktail (TAKARA) and 1× phosphatase inhibitor (TAKARA) for a duration of 30 minutes. The concentration of total protein was determined using a BCA protein assay kit. Extract 10% of the lysate as the input, and the remaining 90% of the lysate is added to the 3μg corresponding antibodies, after shaking at 4°C for 1 hour, add Protein A/G agarose beads. Next day, the precipitate was collected and washed three times with PBS. Subsequently, 1×loading buffer was added, heated at 95°C for 5 minutes, and then a western blot experiment was conducted. For the IP experiment, a specific secondary antibody (Abcam, ab131366, USA) will be used to incubate the PVDF membrane with the primary antibody to avoid interference from the heavy and light chains.

**Cell proliferation assay**

For the short-term proliferation assay, A549 and H1299 cells were seeded into 6-well plates with 50,000 cells/well. Cells were digestion using trypsin and counted using hemocytometer under microscope every 24 hours. The data were analyzed using GraphPad Prism 7 software. All experiments were replicated at least three times with n=3 samples per experiment.

**Wound healing assay**

The H1299 and A549 cells were seeded at a concentration of 8×10^5^ cells/well in 6-well plates. After incubating for 24 hours, the cells were scraped using a 200 µL pipette tip. Following two washes with PBS, the plates were supplemented with FBS-free 1640/DMEM medium. Images of the scratches were captured after 24 hours to determine the proportion of healed area. The data were analyzed using GraphPad Prism 7 software. All experiments were replicated at least three times with n=3 samples per experiment.

**Transwell assay**

The Transwell assay was conducted using a 24-well insert transwell plate with an 8μm pore size. The lower chamber was supplemented with 800μl of medium containing 10% FBS. Subsequently, the upper chamber was inoculated with 5×10^4^ H1299 cells and 1×10^5^ A549 cells in 100μl of serum-free medium. After 24 hours of incubation, the medium was aspirated using a pipette gun, and the chamber was subsequently fixed with 4% paraformaldehyde for 15 minutes. Following fixation, it was stained with 1% crystalline violet for another 15 minutes. Finally, the number of migrated cells beneath the upper chamber was quantified under magnification. All experiments were independently replicated at least three times with n=3 samples per experiment.

**Colony formation**

A549/H1299 cells were digested using trypsin and counted. A total of 1×10^3^ cells were seeded into 6-well plate containing the medium with FBS for 2 weeks. The cells were washed using PBS, and the colonies were fixed by 4% paraformaldehyde for 15 minutes and then stained with 1% crystalline violet for 15 minutes. Finally, the number of colonies was counted under a microscope. The data were analyzed using ImageJ and GraphPad Prism 7 software. All experiments were replicated at least three times with n=3 samples per experiment.

**Immunohistochemistry (IHC)**

Tumor samples from both mice and humans were preserved in formalin and subsequently embedded in paraffin. Thin sections (5μm) were prepared, deparaffinized, rehydrated, and stained using hematoxylin and eosin. Then the sections underwent treatment with 3% hydrogen peroxide solution for 10 minutes to inhibit endogenous peroxidase activity. Antigen retrieval was performed using a 10 mM citrate buffer at pH6.0 for 10 minutes. The tissue sections were then treated with 5% bovine serum albumin blocking reagent for 10 minutes to reduce non-specific binding before being incubated overnight at 4°C with either anti-POU3F3 or anti-Ki67 antibodies. After washing the slides twice with PBS, they were exposed to goat anti-rabbit HRP-conjugated secondary antibodies for an hour at room temperature. Finally, the slides were washed again and developed using 3,3’-diaminobenzidine followed by counterstaining with hematoxylin.

**Sequencing data processing**

Total RNA was isolated from negative control and POU3F3 knock out H1299 cells. Sequencing was performed on GUANGZHOU IGE BIOTECHNOLOGY LTD. Filtering, quality assessment, comparative analysis, and gene annotation were conducted. Reads that were less than 20nt in length or contained ambiguous nucleotides were filtered using Trimmomatic (version 0.30). The remaining reads were aligned to the human genome using TopHat (version 2.0.9). Only uniquely mapped reads with a mapping quality score ≥20 was retained for subsequent analysis for each sample.

**ChIP assay**

ChIP assay was conducted according to the protocol of ChIP Assay Kit (Beyotime, P2078, China). In brief, the cells were fixed with 1% paraformaldehyde at 37°C for 10 minutes in an incubator, followed by the addition of a stop solution to halt the reaction. Subsequently, the cells were lysed and sonicated to fragment the cellular DNA. The corresponding antibody was added to the cell lysate and incubated on a shaker at 4°C for one hour. Protein A/G agarose beads were then introduced and left to incubate overnight. The next day, DNA fragments were recovered and enriched using a DNA purification kit, and the enrichment was assessed via qRT-PCR, the primer’s sequence was listed in Table S3.

**Silver staining**

The silver staining assay was conducted using fast silver staining kit (Beyotime, P0017S, China) according to manufacturers’ protocol. In brief, following electrophoresis, the gel was fixed in the fixing solution for 10 minutes and subsequently washed with 30% ethanol followed by double-distilled water. The gel was then immersed in the sensitizing solution for 2 minutes and rinsed twice with double-distilled water. After sensitization, the gel was incubated with the silver staining solution for 10 minutes, after which the developing solution was added until the desired bands became visible. The reaction was halted by adding the stop solution, and the results were documented using a scanner.

**Blue native polyacrylamide gel electrophoresis (BN-PAGE) assay**

To isolate protein complexes from whole cell homogenates in a single step, blue BN-PAGE was employed following a previously detailed procedure[18]. In brief, cellular mitochondria were isolated by different centrifugation as previously reported[19]. The mitochondrial pellet was resuspended in 6-aminocaproic acid buffer, followed by the addition of detergent digitonin and incubated on ice for 10 minutes. The optimal protein-to-digitonin ratio of 6 g/g was employed. The sample was centrifuged at 20,000g for 20 minutes at 4°C. The resulting supernatant was supplemented with 5 μl of 50% glycerol and 6 μl of 5% Coomassie blue G-250 dye, and aliquots of 10–20 μl were loaded onto a 4–16% gradient native gel. Following a 1-hour BN-PAGE run using a BN anode buffer (50 mM Bis-Tris HCl, pH 7.0) and a BN cathode buffer (50 mM Tricaine, pH 7.0, 150 mM Bis-Tris, 0.02% Coomassie blue G-250), the cathode buffer was replaced with a fresh BN cathode buffer lacking Coomassie blue G-250[18a]. Upon completion of the electrophoresis, the subsequent steps proceeded identically to those of immunoblotting or in gel activity assay.

For immunoblotting, used the primary antibodies against subunits of OXPHOS complexes. For complex Ⅰ, used NDUFB8 antibody (Proteintech, 83216-3-RR, 1:1000 dilution). For complex Ⅱ, used SDHA antibody (Proteintech, 14865-1-AP, 1:1000 dilution). For complex Ⅲ, used UQCRC2 antibody (Proteintech, 83667-2-RR, 1:1000 dilution). For complex Ⅳ, used COX5B antibody (Proteintech, 11418-2-AP, 1:1000 dilution). For complex Ⅴ, used ATP5PB (Abmart, PS18106S, 1:1000 dilution).

For in gel activity assay, used complex Ⅰ substrate solution (2mM PH7.4 Tris-Hcl, 0.1mg/ml NADH, 2.5mg/ml nitrotetrazolium blue chloride) to detected the activity of complex Ⅰ. Used complex Ⅱ substrate solution (1M sodium succinate, 2.5mg/ml nitrotetrazolium blue chloride, 250 mM phenazine methosulfate, 5mM PH7.4 Tris-Hcl) to detected the activity of complex Ⅱ. used complex Ⅳ substrate solution (0.5mg/ml diaminobenzidine, 1mg/ml cytochrome c, 50mM PH7.4 phosphate buffer) to detected the activity of complex Ⅳ. used complex Ⅴ substrate solution (35mM Tris, 270mM glycine, 14mM MgSO_4_, 10mM ATP, 0.2% Pb(NO_3_)_2_) to detected the activity of complex Ⅴ. Upon the appearance of indicated the bands, stop reaction with 50% methanol and scan.

**Luciferase reporter assay.**

To evaluate the effect of POU3F3 on ATP5PF expression, the WT promoter or mutant promoters of ATP5PF were inserted behind the firefly luciferase (F-luc) coding region. Both the WT and mutant luciferase reporter plasmids were transfected into POU3F3 overexpressed HEK293 cells, the F-luc and renilla luciferase (R-luc) was assayed by Luciferase reporter gene kit (Beyotime, RG005 and RG016). The R-luc was normalized to assess the efficiency of plasmid transfection. In brief, after transfection of HEK293 cells with the POU3F3 and reporter plasmids for 48 hours, 1×10^6^ cells were harvested. The cells were lysed using lysis buffer on ice for 30 minutes, followed by centrifugation at 4°C at 12,000 rpm for 15 minutes. The supernatant was then transferred to a new centrifuge tube. According to the manufacturer's protocol, the appropriate luciferase substrate and cell lysate were added to a black-bottomed 96-well plate, after which the plate was immediately transferred to a spectrophotometer to measure the fluorescence values.

**Mitochondrial Membrane Potential Measurements**

The mitochondrial membrane potential was assessed by incubating cells with JC-1 (0.1μM, Beyotime, C2006) in 37℃ incubator for 30 minutes. Subsequently, the cells were rinsed twice with cold PBS before being examined using spectrophotometer or an inverted fluorescent microscope. When detecting JC-1 monomers, the excitation light can be set to 490nm and the emission light to 530nm; when detecting JC-1 polymers, the excitation light can be set to 525nm and the emission light to 590nm.

**ATP and lactate Assays**

The cellular ATP and lactate levels in NSCLC cells were assessed with an ATP assay kit (BC0300, Solarbio) and Lactate assay kit (BC2235, Solarbio). In brief, 1 × 10^6^ cells were plated in 6cm dishes. Following overnight incubation, the cells were lysed using lysis buffer. The supernatants were gathered after centrifugation at 12,000 rpm for 20 minutes at 4 °C and the signal was measured using a spectrophotometer. Total protein lysates were obtained and the protein concentration was determined for normalization using the BCA Protein Assay Kit (KGB2101, KeyGENbioTECH).

**ATP synthase activity assay**

Collected 1×10^6^ cells from different samples and added homogenization buffer (225 mM mannitol, 75 mM sucrose, 0.1 mM EDTA, and 10 mM Tris HCl pH 7.2). The cells were lysed using a homogenizer, followed by centrifugation at 12,000 rpm for 10 minutes at 4°C. The supernatant was transferred to a new pipette. The microplate reader was preheated to 37°C. Meanwhile, assay buffer (0.4 mM NADH, 0.4 mM NADPH, 1 mM PEP, 10 units LDH, 25 units PK, 0.01% w/w DDM, 3 µM AP5A) and the cell lysates were added to a 96-well plate. After a 3-minute reaction in the microplate reader at 37°C, 1 mM ATP was added to start the reaction. Continuous readings were taken at a wavelength of 340 nm for 30 minutes, and the data were recorded.

**Immunofluorescence**

Immunofluorescence was used to evaluate POU3F3 translocation. In brief, cells were grown on sterilized coverslips and fixed with 4% paraformaldehyde for 15 minutes after overnight incubation. Subsequently, the cells were rinsed with PBS three times, permeabilized with 0.1% Triton X-100 at 37℃ incubator for 15 minutes, and then blocked with 0.5% bovine serum albumin for 1 hour. The coverslips with cells were then incubated overnight with a Rabbit monoclonal POU3F3 primary antibody (Proteintech, 18999-1-AP, 1:1000 dilution), followed by incubation with corresponding fluorescence-conjugated secondary antibodies diluted in blocking buffer for 1 hour. Finally, the coverslips with cells were mounted using mounting medium containing DAPI, and images were captured using confocal laser scanning microscopy.

**In vitro phosphorylation assay**

To confirm POU3F3 is a direct downstream target of ERK, we conducted in vitro phosphorylation experiments. First, we purified Flag-POU3F3 and HA-ERK proteins from HEK293 cells. Then, we prepared an in vitro phosphorylation reaction system according to the following formula: 20 mM TRIS-HCl pH 7.4, 150 mM NaCl, 1 mM DTT, 20 mM MgCl2, 0.2 mM ATP. The reaction was incubated at 37°C for 30 minutes, followed by the addition of 1× loading buffer for Western blot analysis.

**Oxygen consumption rate analysis**

Cells were seeded to 10cm dish for 2 days and collected. 1×10^6^/mL cells were added to Oroboros Oxygraph-2k system, and measured the oxygen consumption rate according to manufacturer’s protocol. In brief, different drugs were titrated to the chamber in turn, recorded the data until the curve to plateau.

**Xenograft studies**

Three-weeks-old female BALB/c-nude mice were purchased from GemPharmatech and fed in SPF level barrier system of the laboratory animals science department of Jinan University. 6×10^6^ negative control V2 and POU3F3 knock out cells were subcutaneously inoculated into the right shoulders. Mice were sacrificed and the tumors were collected.

**Gene set enrichment analysis (GSEA)**

GSEA was conducted using version 3.0 of the GSEA software from the Broad Institute[20]. This method assesses gene expression profiles from both negative control and POU3F3 knockout samples, analyzing them in terms of specific gene sets. Normalized enrichment scores (NES) were obtained by performing 1,000 permutations on the genes. A gene set was deemed significantly enriched if it had a normalized p-value less than 0.05 with a false discovery rate (FDR) threshold of less than 0.05 established as the criterion for statistical significance.

**POU3F3 expression and survival prognosis analysis**

NSCLC The Cancer Genome Atlas (TCGA) data were acquired from cBioPortal (https://www.cbioportal.org/)[21]. Expression data of POU3F3 performed with Spearman’s correlation in 659 patient samples including 600 NSCLC patient sample and 59 normal lung epithelial samples, was downloaded also from cBioPortal. The POU3F3 profiles in A549 NSCLC cell line and Beas-2b cell line was obtained from Gene Expression Omnibus (GEO) database (http://www.ncbi.nlm.nih.gov/geo/). For survival prognosis analysis of lung cancer patients, we predicted the impact of high expression of POU3F3 on the overall survival (OS) and progression-free survival (PFS) of lung cancer patients from the Kaplan-Meier Plotter (https://kmplot.com/analysis/)[22].

**Result**

1. POU3F3 was upregulated in lung cancer tissues and cell lines.

Long noncoding RNA POU3F3 (Linc-POUF3F3) has been widely investigated and characterized as an oncogenic Linc-RNA in many types of cancers[23]. Our previous work demonstrated that Linc-POUF3F3 enhances cancer cell proliferation, migration and invasion in NSCLC by downregulating microRNA-30d-5p[24]. Linc-POUF3F3 has been also found to contributes to tumorigenesis by epigenetically modulating POU3F3 gene expression[25]. It is of great interest to evaluate whether the POU3F3 protein participate the cancer development progression. To investigate the role of POU3F3 in NSCLC, we conducted a comparative analysis of its expression levels between 600 NSCLC tissues and 59 paracancerous tissues from TCGA database. Our results demonstrated a significantly increased mRNA level of POU3F3 in NSCLC tissues compared to that of paracancerous tissues (Fig. 1A). Similarly, compared to the normal lung epithelial Beas-2b cell, human NSCLC A549 cell exhibited higher POU3F3 mRNA level (from GEO database GSE160683 and GSE158954) (Fig. 1B). Moreover, Kaplan-Meier analysis displayed that higher POU3F3 mRNA expression is significantly associated with shorter OS and PFS in lung cancer patients (Fig. 1C-D). To further confirm the expression of POU3F3 in NSCLC cells, we performed qRT-PCR and western blot assay to assess the POU3F3 levels in A549 and H1299 cells. The results showed that both mRNA and protein levels are upregulated in these two NSCLC cell lines compared to those in Beas-2b cell (Fig. 1E-F).

To further investigate the expression levels of POU3F3 protein in tumor samples, we conducted an immunoblotting assay to examine the POU3F3 protein expression in NSCLC tissues and corresponding paracancerous tissues from Nanhai Hospital of Traditional Chinese Medicine. The data exhibited that POU3F3 was upregulated in lung cancer tissue compared with that in paracancerous tissue (Fig. 1G). To validate these findings, we performed immunohistochemistry (IHC) staining and found that the expression of POU3F3 was significantly higher in lung cancer tissue than that in paired adjacent normal tissue (Fig. 1H). Together, these data demonstrated that POU3F3 was highly expressed in lung cancer and correlated with the poor prognosis, suggesting that POU3F3 protein expression level is crucial for NSCLC progression and transformation.

1. POU3F3 promoted the proliferation and migration of NSCLC.

To further determined the role of POU3F3 in NSCLC, we established POU3F3 stable knockout cell lines using the CRISPR/Cas9 system (Fig. 2A). A549 and H1299 POU3F3 knockout cells shows slower proliferation and sphere formation compared to their parental cells using cell counting and colony formation assay (Fig. 2B-E), suggesting POU3F3 is important in NSCLC cells proliferation and transformation. To investigate the effect of POU3F3 in cell migration, we performed transwell assay and found the numbers of migratory A549 and H1299 POU3F3 knockout cells in the lower chamber were fewer than those of their parental groups (Fig. 2F-G). Similarly, in wound healing assay, slower migration of POU3F3 knockout A549 and H1299 cells were also observed compared to parental groups (Fig. S1A-B). In addition, to further investigate the role of POU3F3 in NSCLC proliferation in vivo, we performed xenograft experiment using POU3F3 knockout cells and the parental cells. As shown in Figure 2H-I, H1299 POU3F3 knockout cells exhibited decreased tumor mass (Fig. 2H) and Ki67 staining (Fig. 2I), indicating POU3F3 deficiency impaired tumor proliferation capacity in vivo. Altogether, these data demonstr­­­­ated that POU3F3 facilitated the proliferation and migration of NSCLC cells.

1. Knockout of POU3F3 diminishes mitochondrial ATP production in NSCLC cells.

Aerobic glycolysis is a prevalent characteristic of cancer cells[26]. However, emerging evidence shown that OXPHOS also contribute proliferation and metastasis in NSCLC[27]. We next sought to determine whether POU3F3 promote cancer cell proliferation by providing metabolic advantage in NSCLC cells. In our current study, we found cellular lactate is comparable in POU3F3 knockout cells. (Fig. S1C). However, the ATP level was markedly reduced in POU3F3 knockout NSCLC cells compared to that in parental NSCLC cells (Fig. 3A), implying that mitochondrial ATP primarily contributed to the reduction in cellular ATP level response to knockout of POU3F3. To assess the impact of POU3F3 knockout on cellular ATP produced by glycolysis, A549 and H1299 cells were treated with 2-deoxy-D-glucose (2-DG) followed by measurement of ATP levels. Data indicated that 2-DG treatment led to a significant reduction in ATP production in both WT and POU3F3 stable knockout cells (Fig. S1D), suggesting that POU3F3 facilitated ATP production independent glycolysis in NSCLC cells. The mitochondrial ATP synthase utilized the electrochemical gradient of protons across the inner membrane during OXPHOS to catalyze ATP synthesis[27b]. We then determined the mitochondrial membrane potential (MMP) in POU3F3 knockout NSCLC cells. We observed a significant reduction of MMP in POU3F3 knockout cells using inverted fluorescent microscope and spectrophotometry, respectively (Fig. 3B-C). These results suggested that the knockout of POU3F3 lead to the inhibition of cellular MMP and a decrease in ATP production within NSCLC cells. To explore the role of POU3F3 in OXPHOS, we measured the oxygen consumption rates (OCR) linked to ETC complex driven respiration using Oroboros Oxygraph-2k system. As shown in Fig. 3D-E and Fig. S2A-B, POU3F3 knock out hardly damaged basal respiration, but significantly impaired the maximum respiratory potential by inhibiting mitochondrial complexes, including complex I and complex II. The samples were subjected to SDS-Page and analyzed by immunoblotting as loading control (Fig. S2C). To further investigate the effects of POU3F3 knockout on the expression and enzymatic activity of the mitochondrial respiratory complexes (complex Ⅰ，Ⅱ，Ⅲ，Ⅳ and Ⅴ), we conducted BN-PAGE assay, followed by assessing the expression and activity of each complex using silver staining, immunoblotting, and in gel activity assay. Silver staining suggested that the expression level of mitochondrial complex V was markedly reduced in POU3F3 knockout cells (Fig. S2D). Accordingly, immunoblotting showed that the expression of mitochondrial complex V in cells was significantly downregulated, whereas the expressions of complexes I, II, III, and IV remained largely unchanged responding to POU3F3 knockout (Fig. 3F and Fig. S2E). In addition, in gel activity assay showed that knockout POU3F3 decreased the ATP synthase activity while the activities of the other complexes exhibited minimal changes (Fig. 3G and Fig. S2F). In summary, these data confirm that POU3F3 deficiency results in mitochondrial dysfunction, characterized by impaired OXPHOS, decreased ATP synthase expression and activity, and consequently diminished ATP synthesis.

1. Knockout of POU3F3 impaired cellular ATP synthesis through promoting transcription of mitochondrial F_1_F_O_ ATP synthase subunit ATP5PF

Next, we continued to determine the underlying mechanisms by which POU3F3 affect mitochondrial function in NSCLC cells. In consideration of the major function of POU3F3 as a transcription factor, we further explored whether POU3F3 altered mitochondrial function through its downstream effector expression by performing a next-generation mRNA sequencing in POU3F3 knockout H1299 cells. A total of 568 differentially expressed (DE) genes were identified, consisting of 361 upregulated genes and 207 downregulated genes (Fig. 4A). Gene ontology (GO) analysis indicated that downregulated genes were enriched in metabolism process (Fig. 4B). In addition, The GSEA analysis revealed enrichment of different pathways associated with mitochondrial metabolism in the POU3F3 knockout H1299 cells as compared to the parental H1299, including ATP biosynthetic, the ATP metabolism, the ATP biosynthetic coupled electron transport, and the proton motive force driven ATP synthesis (Fig. 4C). These data suggested that POU3F3 knock out impaired the cellular ATP synthesis process. Given that the knockout of POU3F3 inhibits ATP production and mitochondria serve as a primary source of ATP in NSCLC cells, we proceeded to identify potential genes regulating mitochondrial activity among the DE genes. By analyzing overlapping genes between DE genes and human mitochondrial genes, we identified 19 candidates were significantly decreased after knockout of POU3F3 (Fig. 4D and Table S4). To explore the impact of these 19 genes on the proliferation and ATP production of NSCLC cells, we employed small interfering RNA (siRNA, Table S1) to knockdown these 19 genes and determined the proliferation and cellular ATP production. The data demonstrated that among these 19 genes, the knockdown of ATP5PF, which located at the mitochondrial inner membrane and constituted the mitochondrial complex and F_O_ ATP synthase subunits, presented the most conspicuous inhibitory effects on cell proliferation (Fig. 4E) and ATP production (Fig. 4F). These results suggested that POU3F3 enhances cellular ATP production and proliferation by upregulating the expression of ATP5PF.

To further determine the correlation between the expression of POU3F3 and ATP5PF, we examined the expression level of ATP5PF in POU3F3 knock out NSCLC cells. We found that ATP5PF was significantly downregulated at both mRNA and protein levels following the knockout of POU3F3 (Fig. 5A and B). Furthermore, a significant association between the expression level POU3F3 and ATP5PF is also found in NSCLC cell lines from Spearman correlation analysis (Fig. 5C). Given that POU3F3 is a transcription factor, we assumed that POU3F3 regulate the transcription of ATP5PF. We identified 4 potential POU3F3 binding sites (PBS) in ATP5PF promoter region (-2000~0) based on specific binding patterns presumed by the JASPAR database (https://jaspar.genereg.net/) (Fig. 5D). We then conducted a ChIP assay to verify these potential PBS motifs. Data in Figure 5E shown that the sequence of PBS2 and PBS4 but not that of PBS1 and PBS3 were significantly enriched using POU3F3 antibody compared to negative control IgG antibody (Fig. 5E), suggesting the transcriptional factor POU3F3 directly binds to the regions between -1373 to -1360 and -1071 to -1058 sites in ATP5PF promoter. To further explore the regulatory effect of POU3F3 on the transcriptional activity of ATP5PF, we mutant each PBS in turn and carried out luciferase assay by generating pGL3 luciferase reporter plasmids inserted with full length WT fragment（-2000 to 0）or PBS mutant 1-5 of ATP5PF promoter region (Fig. 5F left). We found out that POU3F3 activates ATP5PF transcription in the presence of full length WT fragment of ATP5PF promoter. The fragments with mutation of PBS2 and PBS4, which is referred to as PBS mutant 2 and PBS mutant 4, significantly decreases the luciferase transcriptional activation. Concurrently, the double mutation at the PBS2 and PBS4 sites which are referred to as PBS mutant 5 further diminished the transcriptional activity of luciferase (Fig. 5F right), suggesting both PBS2 and PBS4 are required for ATP5PF transcription. Nevertheless, fragments with mutation of PBS1 and PBS3, referred to as PBS mutant 1 and PBS mutant 3, had scarcely any impact on the transcriptional activation, suggesting PBS1 and PBS3 is dispensable for ATP5PF transcription (Fig. 5F right). These data demonstrated that POU3F3 possibly activates ATP5PF transcription by binding to the regions of -1373 to -1360 and -1071 to -1058 on the ATP5PF promoter.

1. Knockout of POU3F3 downregulated intracellular ATP production by reducing ATP synthase activity.

We then investigated whether the downregulation of ATP5PF consequent to the knockout of POU3F3 led to a decrease of the ATP synthase activity. In vitro enzymatic activity assay was carried out and results revealed that POU3F3 knockout significantly reduced the ATP synthase activity in H1299 and A549 cells compared to the WT (Fig. 6A). These data suggest that POU3F3 enhances ATP synthase activity, thereby increasing ATP production. Since ATP in NSCLC cells was primarily derived from OXPHOS and glycolysis, we sought to explore whether the knockout of POU3F3 downregulated intracellular ATP levels by inhibiting glycolysis. Utilizing an ATP assay kit, we observed that the knockout of POU3F3 indeed reduced the levels of ATP within H1299 and A549 cells (Fig. 6B). However, there was no significant difference in ATP generation between the WT and POU3F3 knockout cells upon treatment with the ATP synthase inhibitor Oligomycin (Fig. 6B). Furthermore, treatment with the glycolytic inhibitor 2-DG further reduced ATP levels in POU3F3 knockout cells (Fig. S1D), indicating that the majority of the ATP downregulation caused by knockout of POU3F3 is attributed to OXPHOS. Collectively, these results proved that POU3F3 primarily promoted the mitochondrial ATP within cells by enhancing the activity of the ATP synthase, rather than regulating ATP synthesis through glycolysis.

The ATP synthase complex is primarily composed of FoF1 subunits, with ATP5PF resembling a peripheral stalk that plays a crucial role in connecting the FoF1 subunits. Based on the structural analysis of the ATP synthase[28], we identified that ATP synthase subunits ATP5PB and ATP5PD may directly interact with ATP5PF (Fig. 6C). Consequently, we performed Co-Immunoprecipitation (Co-IP) experiments to detect the interaction between ATP5PF and ATP5PB and ATP5PD. Flag pulldown in HEK293 cells overexpressing Flag-ATP5PF demonstrated that ATP5PF interacted with ATP5PB and ATP5PD (Fig. 6D). Additionally, endogenous ATP5PB IP experiments in H1299 cells indicated that the binding of ATP5PB with ATP5PD was downregulated following the knockout of POU3F3 (Fig. 6E). Similarly, endogenous ATP5PD IP experiments in H1299 cells also indicated that the binding of ATP5PD with ATP5PB was downregulated upon the knockout of POU3F3 (Fig. 6E). These results suggested that ATP5PF is essential for the connection between ATP synthase subunits ATP5PB and ATP5PD. In summary, POU3F3 upregulates the expression of ATP5PF, which in turn promotes the binding of ATP synthase subunits ATP5PB and ATP5PD, enhancing the activity of the ATP synthase and subsequent ATP production.

1. POU3F3 knockout NSCLC cells expressing ATP5PF rescue proliferation and migration capacities.

To further determine whether ATP5PF is a major downstream effector of POU3F3 that mediated the increased proliferation and migration in NSCLC cells, we examined the cell proliferation and migration in POU3F3 knockout NSCLC cells by expressing ­­Flag-ATP5PF (Fig. 7A-C). Cell counting assay data indicated that cell proliferation was inhibited by POU3F3 knockout, which is consistent with was rescued by overexpression ATP5PF (Fig. 7B). In addition, transwell and wound healing assay proved that the of POU3F3 knockout was reversed by overexpression ATP5PF (Fig. 7C and D). The data above indicated that ATP5PF is a downstream effector of POU3F3 and promotes NSCLC cells proliferation and migration. To explore whether knockout of POU3F3 decreases cellular ATP production through down-regulating the expression of ATP5PF, we rescued ATP5PF in POU3F3 knockout cells and measured ATP production within the cells. The results demonstrated that knockout of POU3F3 decreased the MMP, and overexpression of ATP5PF is capable of enhancing the cellular MMP in POU3F3 knockout cells (Fig. S3A-B). Moreover, overexpression of ATP5PF was capable of rescuing the decreased ATP production resulting from the knockout of POU3F3 (Fig. S3C). Overall, these results demonstrated that POU3F3 promoted NSCLC proliferation and metastasis by upregulating the expression of ATP5PF, thus increasing cellular MMP and subsequent ATP production.

1. KRAS^G12D^ promoted phosphorylation of POU3F3, resulting in its translocation to the nucleus.

One of the most frequently mutated oncogenes in NSCLC is RAS. Notably, 98% of the RAS mutations at one of three mutational hotspots: G12, G13 and Q61. To investigate the potential role of RAS mutant in modulating POU3F3, we assessed the protein expression and phosphorylation level of POU3F3 in H1975, H522, PC9 (RAS WT) and H460, A549, H1299 (RAS mutant) cells, results suggested that the phosphorylation of POU3F3 was upregulated in RAS mutant cells, along with the comparative expression level of POU3F3 (Fig. S4A). Meanwhile, data shows that both the activity of ATP synthase and the ATP levels were significantly elevated in RAS mutant cells compared to RAS wild type cells (Fig. S4B). The results suggest that RAS mutation may promoting ATP level via phosphorylating POU3F3. In NSCLC, KRAS mutations accounted for 93% of all RAS mutations, with KRAS^G12^ mutations constituting 95% of all KRAS mutations[29]. This phenomenon underscored the pivotal role of KRAS^G12^ mutation in the pathogenesis of NSCLC. To validated the role of G12 mutant KRAS in promoting the phosphorylation of POU3F3, we ectopically expressed KRAS^G12D^ in H1299 (KRAS WT) cells and subsequently assessed the phosphorylation status of POU3F3. The western blot results demonstrated that upregulation of KRAS^G12D^ leads to an elevation in the phosphorylation status of POU3F3, which was reversed by RAS inhibitor RMC-7977[30] (Fig. 8A). Furthermore, we confirm that RMC-7977 specifically inhibits mutant RAS, while exhibiting minimal inhibitory effects on WT RAS in PC9 cells (Fig. S4C). Additionally, RMC-7977 inhibited POU3F3 phosphorylation in RAS mutant cells A549 and H1299 but not in RAS WT cell H1975 (Fig. S4D). These results suggest that KRAS^G12D^ promotes the phosphorylation of POU3F3, and RMC-7977 in turn restrains POU3F3 phosphorylation by inhibiting KRAS^G12D^. As POU3F3 was role as a transcription factor crucial for nuclear localization and downstream gene transcription, we then investigated the impact of phosphorylation on POU3F3 nuclear localization. Immunofluorescence experiments revealed that the ectopically expressed KRAS^G12D^ resulting the nuclear translocation of POU3F3 (Fig. S4E). The result was further confirmed by a subcellular fractionation assay (Fig. 8B). These findings suggested that the KRAS^G12D^ mutation enhanced the phosphorylation of POU3F3, resulting in its translocation to the nucleus. To investigate the potential interaction between the downstream effector ERK1/2 of the RAS signaling pathway and POU3F3, which may lead to the phosphorylation of POU3F3, Co-IP assay was conducted. The endogenous immunoprecipitation experiment revealed the interaction between POU3F3 and ERK1, and overexpressing of ERK1 promoted the phosphorylation level of POU3F3 (Fig. 8C). Furthermore, subcellular fractionation followed by Co-IP experiments demonstrated that POU3F3 interacts with ERK1 in the cytoplasm rather than in the nucleus (Fig. S4F). These results indicate that POU3F3 interacts with ERK1 in the cytoplasm. To demonstrate that POU3F3 is a direct downstream substrate of ERK1, we carried out in vitro phosphorylation assays. The results indicated that ERK1 could directly facilitate the phosphorylation of POU3F3 (Fig. 8D). We next performed mutational analysis and generated 7 phospho-deficient T/A and S/A mutants of POU3F3 based on the canonical phosphorylation S/T-P motif of ERK [31]. Result shows that substitution of S393 with alanine resulted in abolishment of ERK1-dependent phosphorylation of POU3F3 (Fig. 8E). These data indicates that ERK1 phosphorylates POU3F3 at S393 site in cell cytoplasm. We next investigated whether ERK1 mediates KRAS^G12D^-induced nuclear localization of POU3F3. Subcellular fractionation and immunofluorescence assay exhibit that overexpressed 6×HA-ERK1 promotes the nuclear translocation of POU3F3 (Fig. 8F and Fig. S4G). To further verify that ERK1 promotes the nuclear localization of POU3F3, we treated the cells with the specific ERK1 phosphorylation inhibitor SCH772984. Western blot analysis showed that SCH772984 significantly decreased the nuclear localization of POU3F3 in both A549 and H1299 cells (Fig. S4H). In addition, we found that SCH772984 effectively inhibited POU3F3 phosphorylation and was accompanied by a downregulation of ATP5PF expression (Fig. S4I). All these results suggest that ERK1 promotes the nucleus translocation of POU3F3, which promotes the transcription of ATP5PF.

The importins are part of a conserved group of mobile receptors responsible for facilitating the movement of large molecules across the nuclear membrane. To further elucidated the mechanism of POU3F3 nuclear translocation, we conducted a screening for transporters responsible for mediating POU3F3 nuclear localization by employing CRISPR/Cas9 (sequence of sgRNAs are listed in Table S1) to knockout importin family proteins. Results showed that knockout importin β1 significantly decreased the nuclear localization of POU3F3 (Fig. 8G and Fig. S4J). Instead, overexpression of importin β1 promoted POU3F3 nuclear localization (Fig. S4J), suggesting that importin β1 is responsible for mediating the nuclear translocation of POU3F3. Moreover, we found that there is an interaction between POU3F3 and importin β1, and RMC-7977 can suppress the binding between them (Fig. 8H). Altogether, our experiments demonstrated that ERK1 directly bind to and phosphorylated POU3F3 at S393 site in the cytoplasm, facilitates its interaction with importin β1 and leads to its nuclear translocation, thereby elevating the expression of ATP5PF and the activity of ATP synthase, subsequently upregulating the ATP production.

In summary, our results found that POU3F3 was upregulated in NSCLC tissue compared with paracancerous tissue, accelerating the proliferation of NSCLC in vivo and in vitro. Mechanically, we demonstrated that POU3F3 bind to ATP5PF promoter (-1373 to -1360 and -1071 to -1058) and accelerating its transcription, thus upregulating ATP synthase activity and subsequent ATP production. Moreover, we had demonstrated that ERK1 promoted the phosphorylation level of POU3F3 at S393 site, concomitant with an accumulation in nuclear localization, thus initiating ATP5PF transcription (Fig. 9).

**Discussion**

POU domain proteins represent a group of eukaryotic transcription factors with highly preserved homeodomains. The previous studies had demonstrated that POU-domain containing family proteins were predominantly expressed in the nervous system and play a role in neurodevelopment[32]. Nevertheless, recent research has demonstrated that POU family proteins are highly expressed in tumor cells and are closely related to tumorigenesis [33]. For instance, POU3F2 regulated cellular differentiation, and contributed to tumor progression in different kind of tumor, such as cell lung cancer[34], melanoma[35] and glioblastoma[36]. In addition, POU2F1 is upregulated in head and neck squamous carcinoma[37], and is important for stemness of cancer stem cell[38]. However, as a member of POU-domain containing protein family, the biological function and underlying mechanism of POU3F3 in NSCLC are still to be discovered. Our data revealed that POU3F3 was significantly upregulated in both NSCLC tissues and cell lines. Mechanically, we found that POU3F3 enhanced the transcription of ATP5PF by binding its promoter region, resulting in elevated ATP5PF expression and ATP production, thereby facilitating the progression of NSCLC. Similarly, previous study demonstrated a positive correlation between MTA1 and the expression of ATP5A, MTA1 interact with ATP5A to accelerate ATP production[27a]. These findings suggested that aberrant expression or activity of ATP synthase subunits, contributed to the development of tumors. Nevertheless, it was imperative to explore whether POU3F3 regulated the functionality of ATP synthase independent of ATP5PF expression.

The "Warburg effect" is commonly acknowledged as the primary theory for the reprogramming of glucose metabolism particularly in cancer. It is anticipated that there will be an increase in glycolysis and a decrease in OXPHOS in the presence of ample oxygen[39]. Nevertheless, recent studies indicated that OXPHOS and glycolysis collaborate to maintain the balance of cellular energy metabolism, mounting evidence indicating the pivotal role of OXPHOS in facilitating tumor proliferation and metastasis[8, 40]. For example, certain tumors including NSCLC rely heavily on OXPHOS for ATP production[41]. Hence, in addition to aerobic glycolysis, the contribution of ATP provided by OXPHOS to tumors should not be overlooked. Nevertheless, the molecular mechanisms underlying the promotion of tumor progression by OXPHOS remain to be elucidated.

KRAS mutation represents the most prevalent genetic mutation in NSCLC and serves as a pivotal driving factor in the pathogenesis of NSCLC[42]. Despite the gradual development of KRAS inhibitors, clinically targeted therapies against KRAS have not demonstrated prominent efficacy[43]. Additionally, Alternative strategies for targeting KRAS have been constrained by adverse effects resulting from non-specific engagement with WT KRAS [44]. Thus, a better understanding of RAS signaling results in the creation of potentially effective inhibitors for KRAS-mutant tumors. It is noteworthy that recent research suggested that KRAS mutations enhance the activity of OXPHOS in tumor cells. The OCR indicative of the extent of mitochondrial OXPHOS, showed a significant increase in KRAS mutation pancreatic cells[45]. Moreover, cells harboring KRAS mutations exhibited heightened susceptibility to OXPHOS inhibitors[46], instead, KRAS mutations diminished the sensitivity of NSCLC to MEK inhibitors by inducing activation of the OXPHOS[47]. These findings implied that KRAS mutations may promote the progression of NSCLC by enhancing the activity of OXPHOS. Consistent with the above, we observed that overexpressing the KRAS^G12D^ mutant enhanced the phosphorylation level of POU3F3 and its nuclear translocation by promoting the interaction between ERK1 and POU3F3. Our findings offered insight into the molecular mechanism through which KRAS mutations driven the development of NSCLC by elevating cellular OXPHOS levels. Nevertheless, challenges still persisted in the clinical trials regarding resistance to KRAS inhibitors. Further exploration to elucidate the involvement of POU3F3 in conferring resistance in NSCLC cells is necessary.

In current study, our findings revealed that phosphorylation of POU3F3 promotes enhanced mitochondrial ETC and coupled ATP production to contribute KRAS^G12D^ NSCLC proliferation. All these results provide new insights into the mechanism of energy balance associated with the "Warburg effect." In fact, several drugs targeting cellular OXPHOS are currently in development, and initial research findings indicate that specific approaches may hold promise for clinical efficacy[48]. The findings collectively indicate that exerts a pivotal role in tumor progression, underscoring the potential of targeting OXPHOS for clinical therapy.

**Conclusion**

Our study demonstrates the oncogene POU3F3 is overexpressed in NSCLC tissues and cell lines, high POU3F3 expression indicates an unfavorable prognosis for the lung cancer patients. We confirmed that POU3F3 promotes NSCLC proliferation in vivo and in vitro. Mechanically, POU3F3 binds to the promoter region -1373 to -1360 and -1071 to -1058 site of ATP5PF, hence initiating transcription of ATP5PF. Elevated expression of ATP5PF facilitates proliferation and migration of NSCLC by promoting OXPHOS and ATP production. Moreover, we have identified POU3F3 as a downstream target of the RAS-RAF-MEK-ERK cascade. The KRAS^G12D^ mutation enhances the interaction between ERK1 and POU3F3, resulting in an elevation of POU3F3 phosphorylation levels and subsequent translocation into the nucleus. Our study, focusing on energy metabolism, elucidated the role of POU3F3 in facilitating the proliferation and migration of NSCLC, thus establishing a theoretical basis for understanding the pathological progression of NSCLC.

**Supporting Information:** Supplementary Figure S1, Figure S2, Figure S3 and Figure S4; Supplementary Table S1, Table S2 Table S3 and Table S4. List of the primer sequences used in our studies; List of the sgRNA sequences used in our studies. Supporting Information is available from the Wiley Online Library or from the author.

**Author Contributions:** Yong Dai，Jun Fan and Dan-Yang Zhou managed the project and wrote the paper; Qi-gang Zeng, Le Li, Tao Chang, Bin Zheng performed the majority of the experiments. Yong Sun, Ling-Na Xue, Chao-Ling Liu Xia-Qing Li, Jia-Xin Gu, Zhao-Rong An, Hao-Tao Yao performed all the other experiments. Ruo-Tong Huang provided critical reading & editing for the manuscript. Yong Dai supervised the research and interpreted the data.

**Funding:**

1. Subsidy funds for the Central Financial Medical Service and Security Capacity Enhancement Project (Inheritance and Development of Traditional Chinese Medicine, Z155080000004)

2. Guangdong Province Key Discipline of Traditional Chinese Medicine (Clinical) Construction Project (No. 1002042801)

3. National Natural Science Foundation Project (No. 81974430)

4. Major Talent Program of Guangdong Provincial (2019QN01Y490)

5. Undergraduates Innovation Programme of Jinan University (202310559035, S202410559139)

6. Guangdong Natural Science Foundation Project (2019A1515012037, No. 2023A1515010578)

**Conflict of interest statement:** The author declared no conflict of interest.

**Acknowledgement:** The authors thanks to School of Medicine of Jinan University for providing access to essential research facilities, including spectrophotofluorometer and Oroboros instruments, as well as for their invaluable technical support.


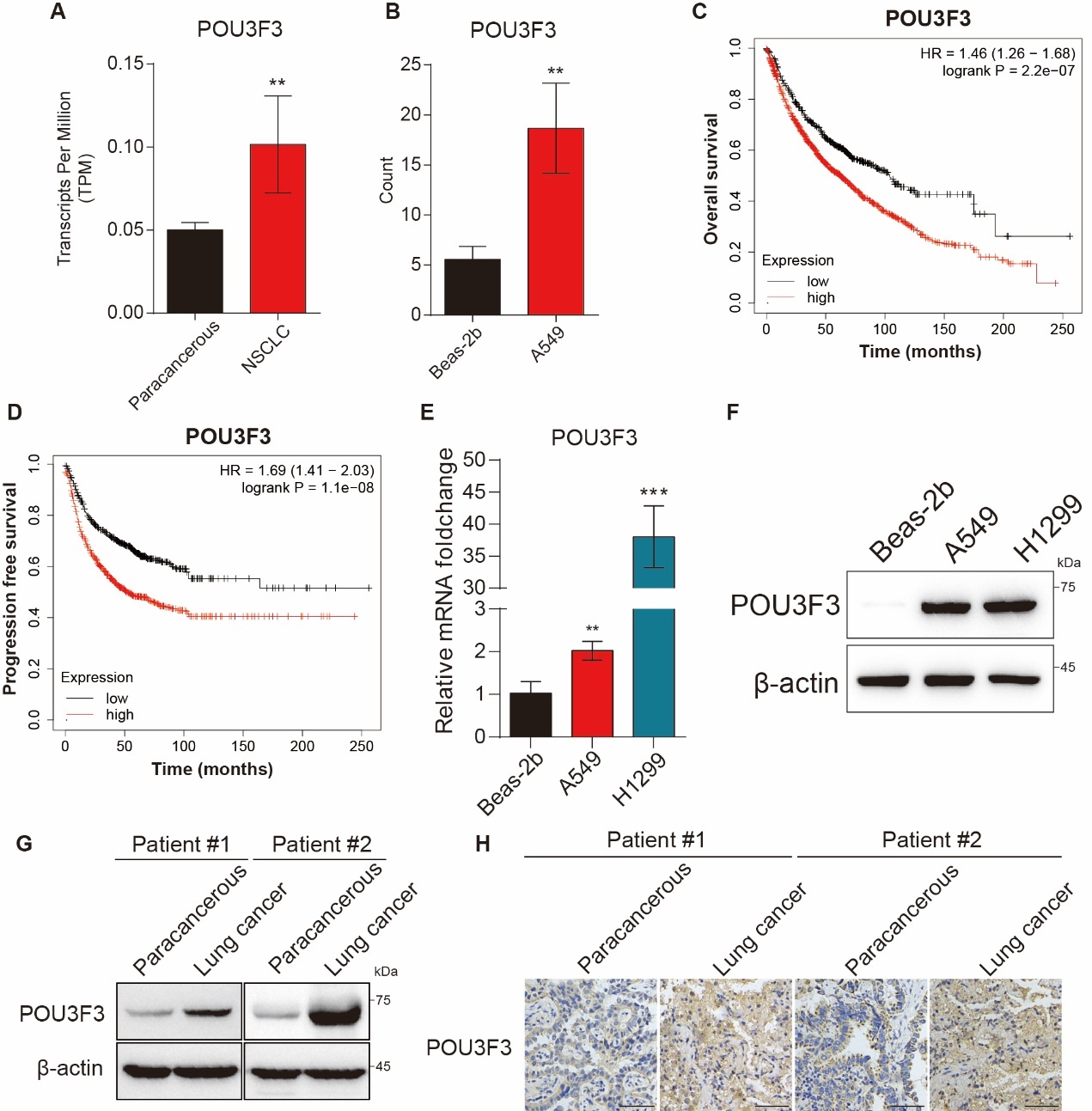


Figure 1. POU3F3 was upregulated in lung cancer tissue. A. POU3F3 mRNA expression was upregulated in NSCLC and normal tissues in TCGA database. Data are mean ± SD, n=3 each, p-value were obtained by two-tailed Student’s test. B. POU3F3 expression was upregulated in NSCLC A549 and normal lung epithelial Beas-2b cell lines. Data are mean ± SD, n=3 each, p-value were obtained by two-tailed Student’s test. C and D. Kaplan-Meier analysis of OS and PFS in high- and low-expressed POU3F3 NSCLC patients. E. The mRNA level of POU3F3 was upregulated in NSCLC cell lines A549 and H1299 cell lines compared to normal lung epithelial cell line Beas-2b. Results represent three independent experiments and are presented as mean ± SD (⁎p < 0.05, ⁎⁎p < 0.01, ⁎⁎⁎p < 0.001 vs. control group). F. The expression level of POU3F3 was upregulated in NSCLC cell lines A549 and H1299 cell lines compared to normal lung epithelial cell line Beas-2b. G. The mRNA level of POU3F3 was upregulated in NSCLC tissue compared to paracancerous tissue. H. The expression of POU3F3 was determined by IHC assay in lung cancer tissues and paracancerous tissue.


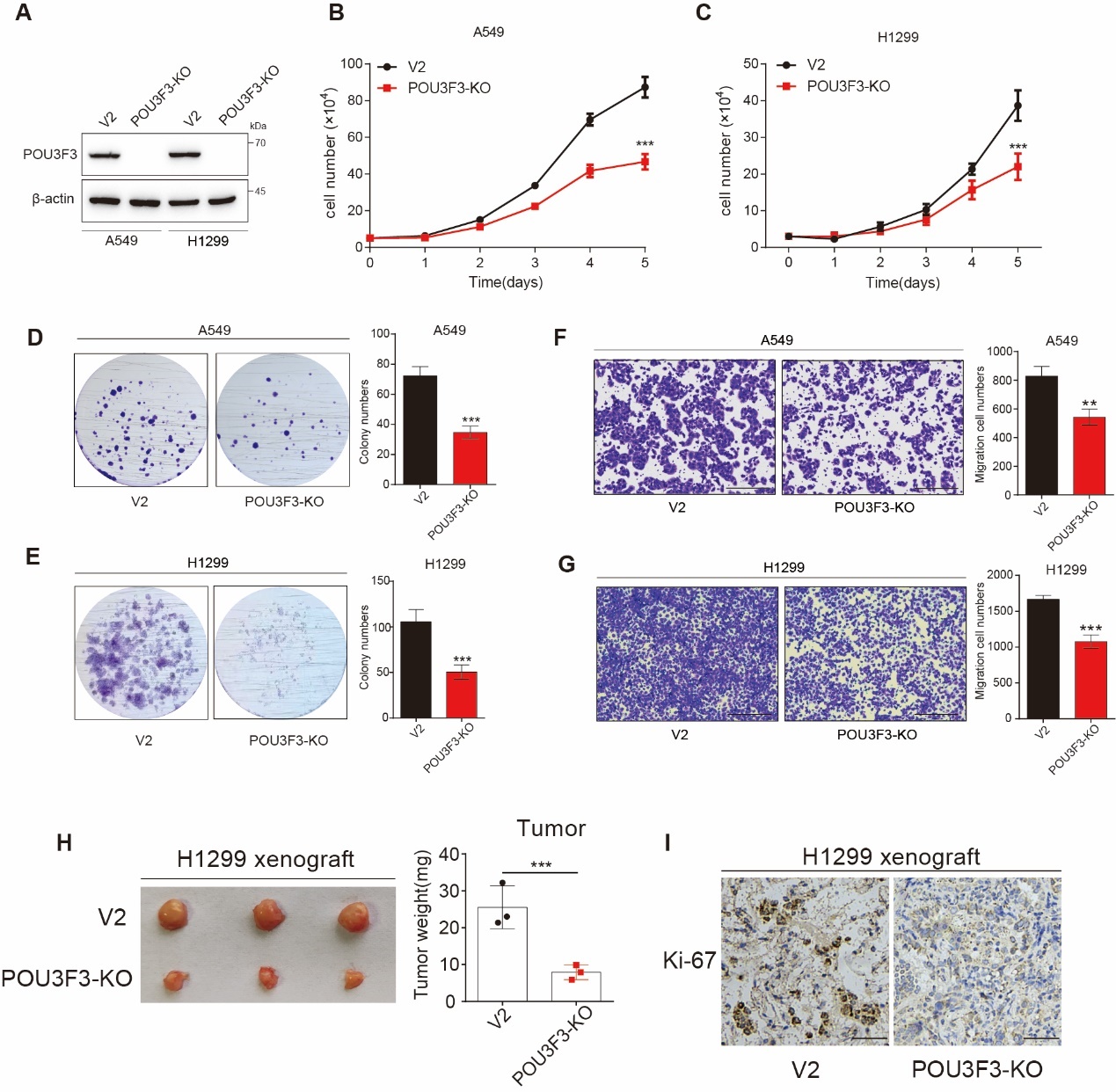


Figure 2. POU3F3 promoted the proliferation and migration of NSCLC. A. Western blot analysis of the titer of sgRNA targeting POU3F3. B and C. Stably knock out POU3F3 suppressed cell proliferation of A549 and H1299 cells. Data are mean ± SD, n=3 each, p-value was obtained by two-tailed Student’s test. D and E. Stably knock out POU3F3 suppressed colony formation of A549 and H1299 cells. The results are based on three independent experiments and are shown as the mean ± SD (⁎p < 0.05, ⁎⁎p < 0.01, ⁎⁎⁎p < 0.001 vs. the control group). F and G. Stably knock out POU3F3 suppressed migration of A549 and H1299 cells. The results are based on three independent experiments and are shown as the mean ± SD (⁎p < 0.05, ⁎⁎p < 0.01, ⁎⁎⁎p < 0.001 vs. the control group). H. Tumor volume and weight of nude mice with H1299 cells in xenograft model. Data are mean ± SD, n=3 each, p-value were obtained by two-tailed Student’s test. I. The expression of Ki-67 was determined by IHC assay in xenograft tissue.


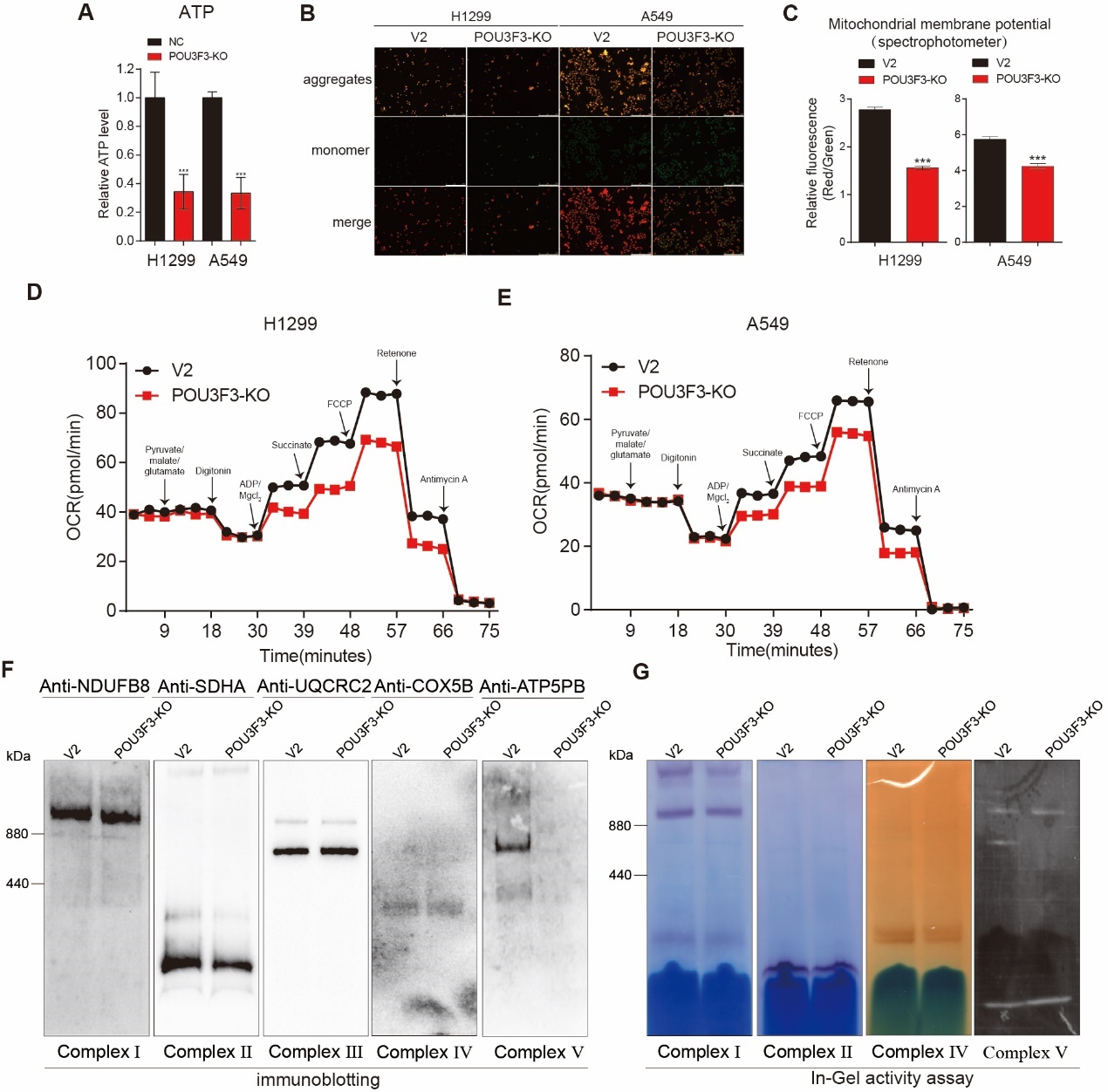


Figure 3. Knockout of POU3F3 diminishes mitochondrial ATP production through inhibiting the respiratory chain in NSCLC cells. A. Knockout of POU3F3 decreased cellular ATP production. The results are based on three independent experiments and are shown as the mean ± SD (⁎p < 0.05, ⁎⁎p < 0.01, ⁎⁎⁎p < 0.001 vs. the control group). B. Inverted fluorescent microscope images displayed lower MMP in POU3F3 knockout cell compared to negative control cells. C. Spectrophotometer exhibited lower MMP in POU3F3 knockout cell compared to negative control cells. The results are derived from three independent experiments and presented as the mean ± SD. D and E. Effects of stably knockout of POU3F3 on mitochondrial respiration of H1299 (D) and A549(E) cells. The results are derived from three independent experiments and presented as the mean ± SD. F. BN-PAGE followed by immunoblotting assay exhibited that knockout POU3F3 decreased the formation of mitochondria complex Ⅴ. G. BN-PAGE followed by in gel activity assay showed that knockout of POU3F3 inhibited the activity of mitochondria complex Ⅴ.


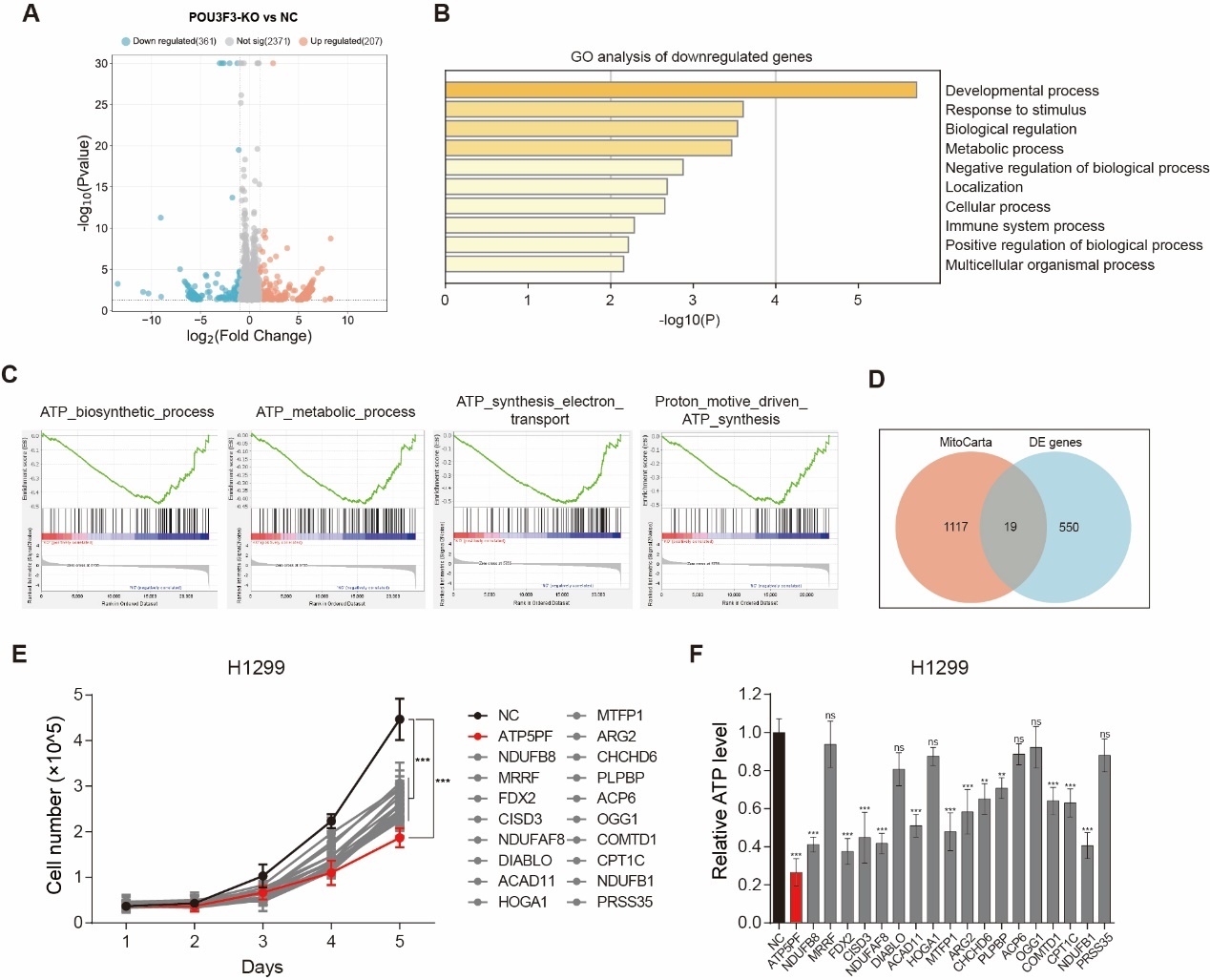


Figure 4. POU3F3 promoted OXPHOS through upregulating the activity of mitochondrial ATP synthesis processes. A. Volcano plot of DE genes between negative control and POU3F3 knock out H1299 cells (POU3F3 vs NC, |logFC| > 1.5, p < 0.05). B. GO analysis of downregulated genes. C. GSEA suggested the association between POU3F3 and cellular metabolism process. D. Overlapping between DE genes and mitochondrial relative genes. E. Knockdown of specified 19 mitochondrial genes suppressed cell proliferation of H1299 cells. Data are mean ± SD, n=3 each, p-value were obtained by two-tailed Student’s test. F. Knockdown of specified 19 mitochondrial genes suppressed cellular ATP production of H1299 cells. Data are mean ± SD, n=3 each, p-value were obtained by two-tailed Student’s test.


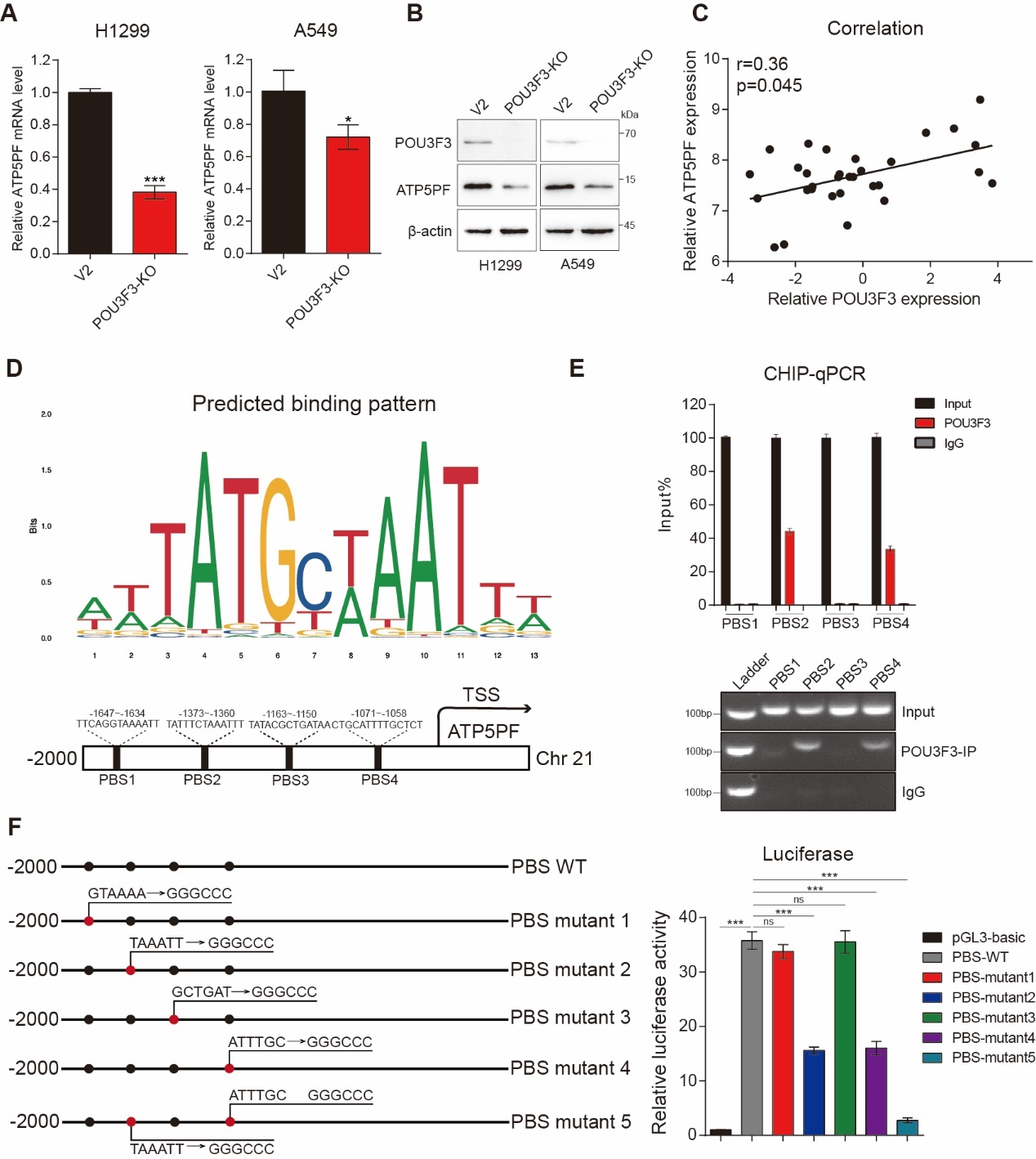


Figure 5. POU3F3 directly bound to ATP5PF promoter and induced its expression. A. Knockout of POU3F3 decreased the mRNA expression of ATP5PF in H1299 and A549 cells. The results are based on three independent experiments and are shown as the mean ± SD. B. Knockout of POU3F3 decreased the expression of ATP5PF in H1299 and A549 cells. C. Correlation analysis between POU3F3 and ATP5PF. D. Sequence and potential POU3F3 binding sites of the core promoter region of ATP5PF. E. H1299 cell line was conducted with a ChIP assay. Anti-POU3F3 and Anti-IgG (negative control) were used to precipitate proteins bound to the amplified sequence of the endogenous ATP5PF promoter in vivo. Results represent three independent experiments and are presented as mean ± SD (*p < 0.05, ⁎⁎p < 0.01, ⁎⁎⁎p < 0.001 vs. control group). F. Luciferase reporter assay for the mutant PBS sequence in HEK293 cells following POU3F3 overexpression.


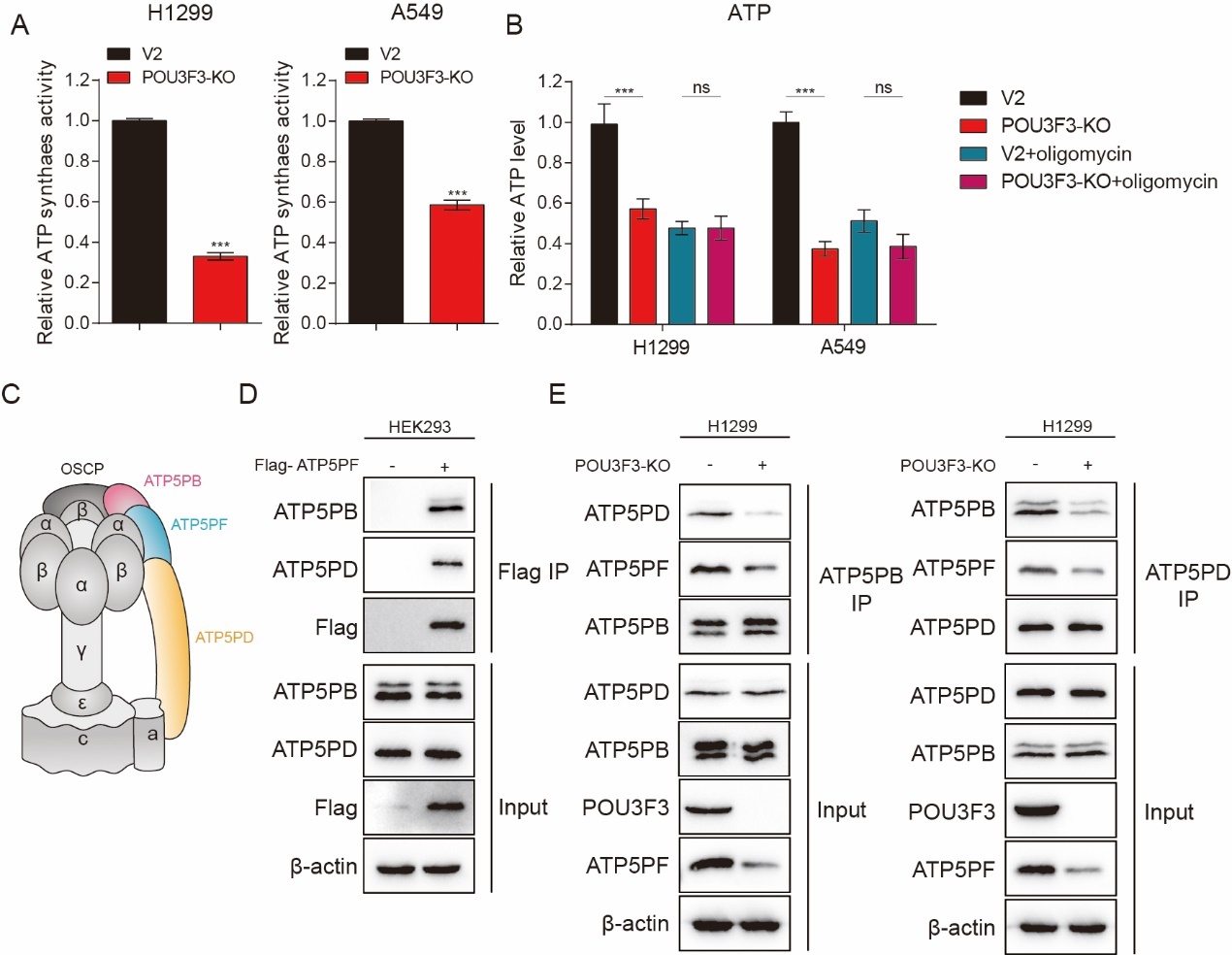


Figure 6. Knockout of POU3F3 downregulated intracellular ATP production by reducing ATP synthase activity. A. Relative ATP synthase activity in WT and POU3F3 knockout H1299 and A549 cells. B. Relative ATP production in WT and POU3F3 knockout H1299 and A549 cells treated with or without ATP synthase inhibitor oligomycin. C. Schematic of the distribution of ATP synthase complex, which are highlighted with different colors on ATP5PF and the subunit associated therewith ATP5PB and ATP5PD. D. Flag pulldown and western blot to detect the interaction between ATP5PF and ATP5PB and ATP5PD. E. Western blot detected the interaction between ATP5PB and ATP5PD in WT and POU3F3 knockout cells.


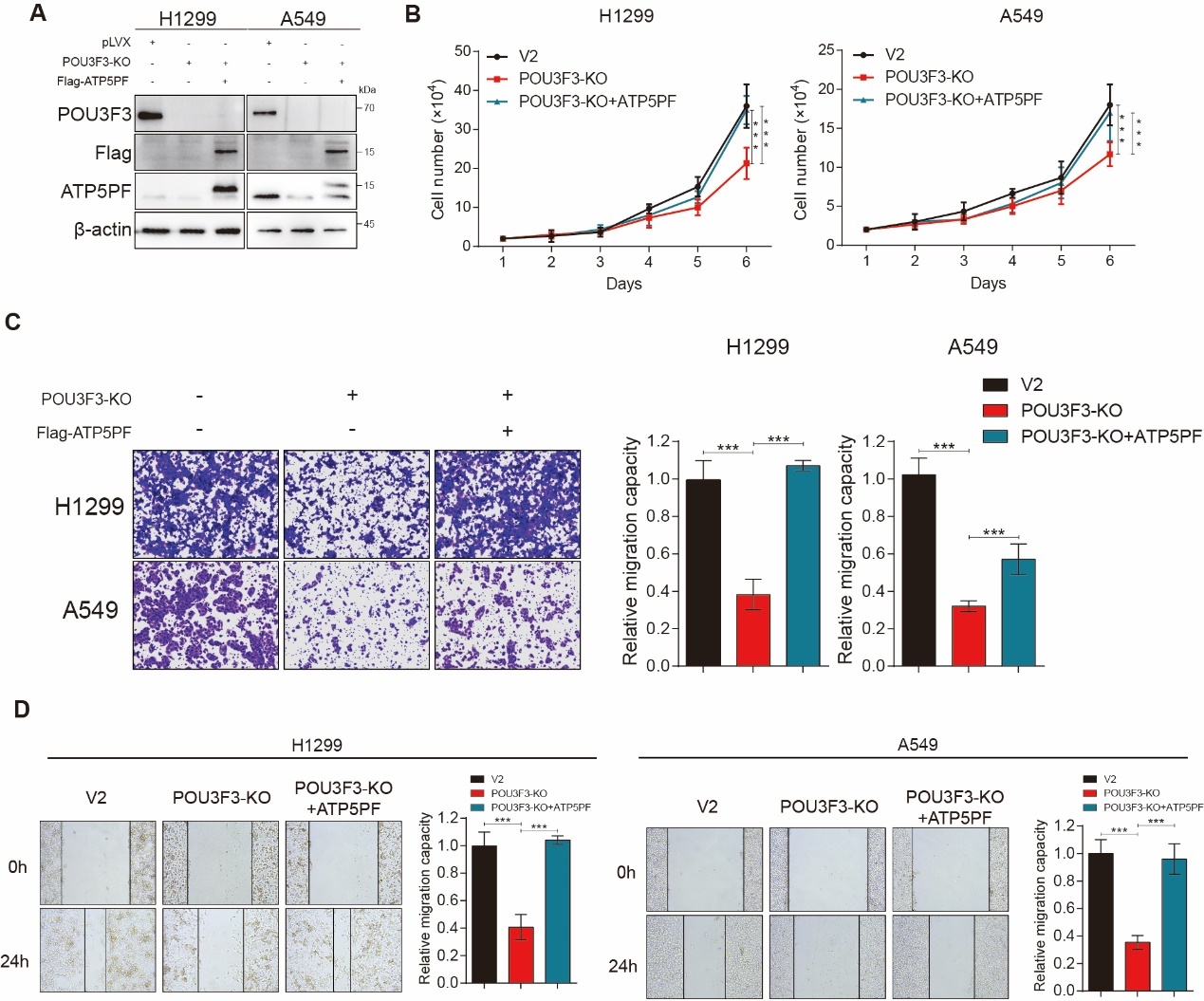


Figure 7. POU3F3 knockout NSCLC cells expressing ATP5PF rescue proliferation and migration capacities. A. Relative protein level of POU3F3 and downstream ATP5PF in WT and POU3F3 knockout H1299 and A549 cells. B. Cell proliferation assay of POU3F3 knockout A549 and H1299 cells stably expressing ATP5PF. C. Transwell migration assay of POU3F3 knockout A549 and H1299 cells stably expressing ATP5PF. D. Wound healing assay of POU3F3 knockout A549 and H1299 cells stably expressing ATP5PF. Results represent three independent experiments and are presented as mean ± SD (*p < 0.05, ⁎⁎p < 0.01, ⁎⁎⁎p < 0.001 vs. control group).


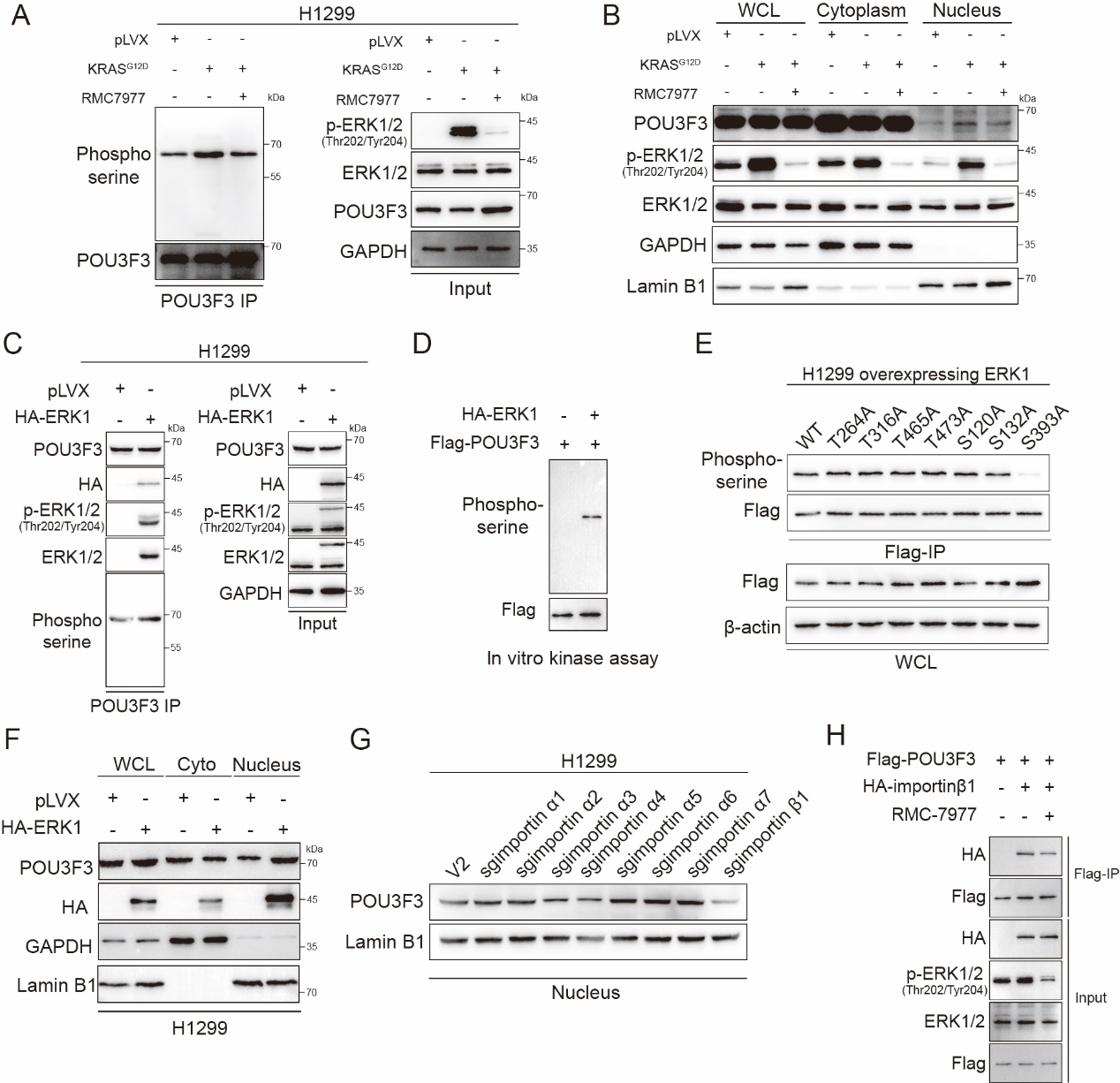


Figure 8. KRAS^G12D^ promoted phosphorylation of POU3F3, resulting in its translocation to the nucleus. A. Overexpression of KRAS^G12D^ promoted the phosphorylation of POU3F3. B. Subcellular fractionation exhibited KRAS^G12D^ translocated POU3F3 into nucleus. C. Co-IP experiment proved that ERK1 interacted with POU3F3 and enhanced its phosphorylation. D. Purified Flag-POU3F3 was incubated with activity ERK1 followed by western blot to verify POU3F3 was a specific substrate of ERK1. E. Overexpressed Flag-POU3F3 WT and each mutant followed by Co-IP and western blot to confirm S393 was phosphorylation site of POU3F3. F. Subcellular fractionation of ectopically expressed 6×HA-ERK1 cells to confirm ERK1 promoting POU3F3 nucleus translocation. G. After sequentially knockout importin family proteins and performing western blot analysis, confirmed that importin β1 mediates the nuclear localization of POU3F3. H. Treated cell with RMC-7977 followed by western blot suggests the phosphorylation of POU3F3 enhances its nuclear localization.


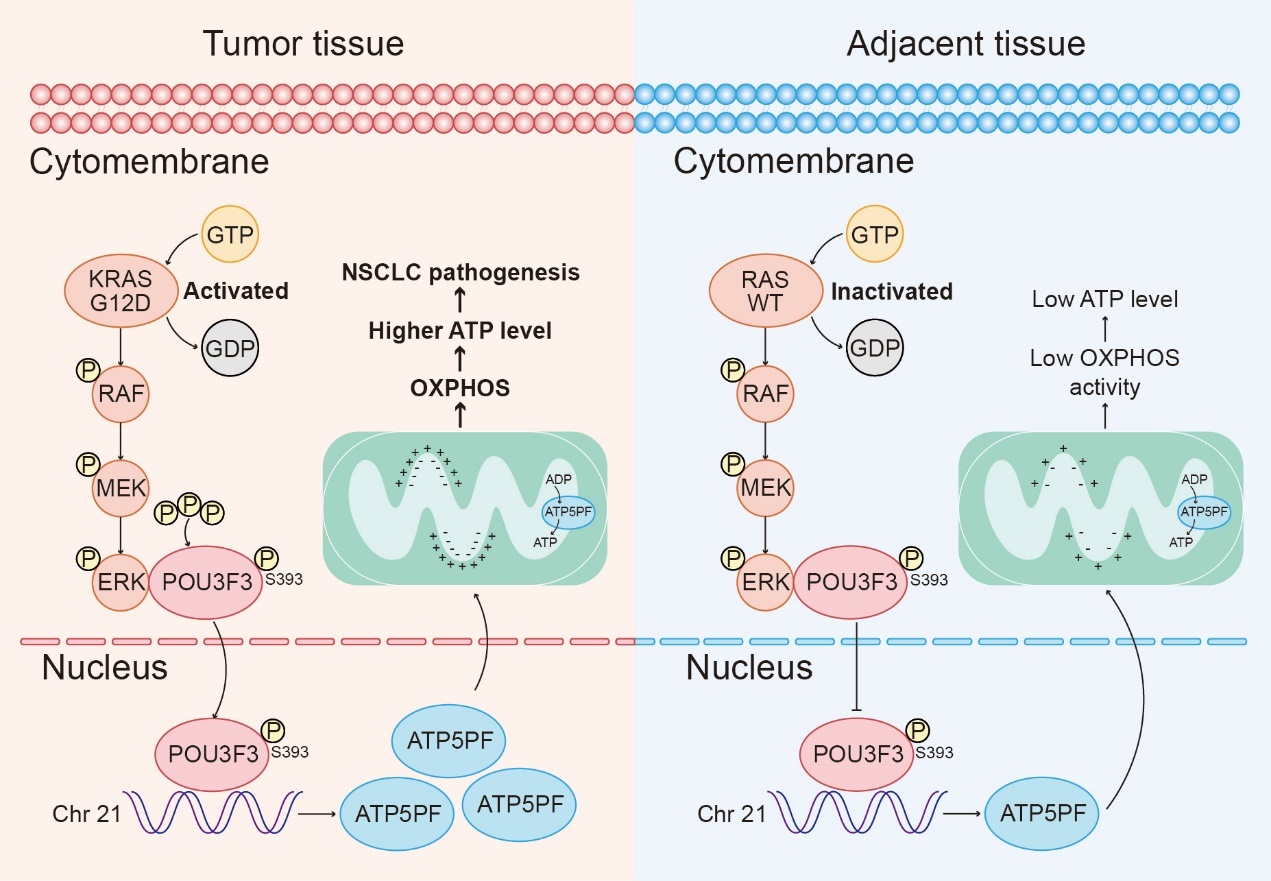


Figure 9. Schematic diagram showing the mechanism of POU3F3 promoted NSCLC progression by promoting OXPHOS and ATP production via elevated the transcripts of ATP5PF.


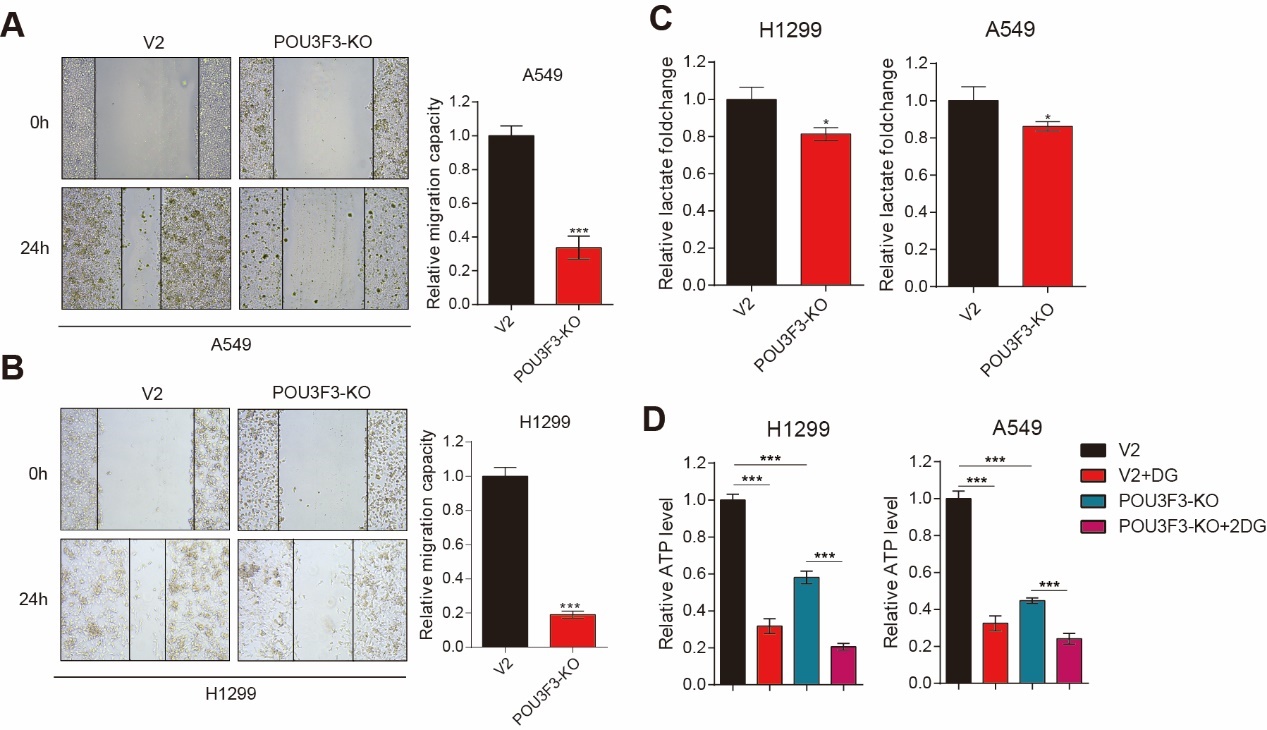


Figure S1. A and B. Wound healing assay displayed stably knock out POU3F3 impaired migration of A549 and H1299. C. Knockout of POU3F3 inhibited cellular lactate production. D. Relative ATP level in WT and POU3F3 knockout cells treated with or without 2-DG. Results represent three independent experiments and are presented as mean ± SD (*p < 0.05, ⁎⁎p < 0.01, ⁎⁎⁎p < 0.001 vs. control group).


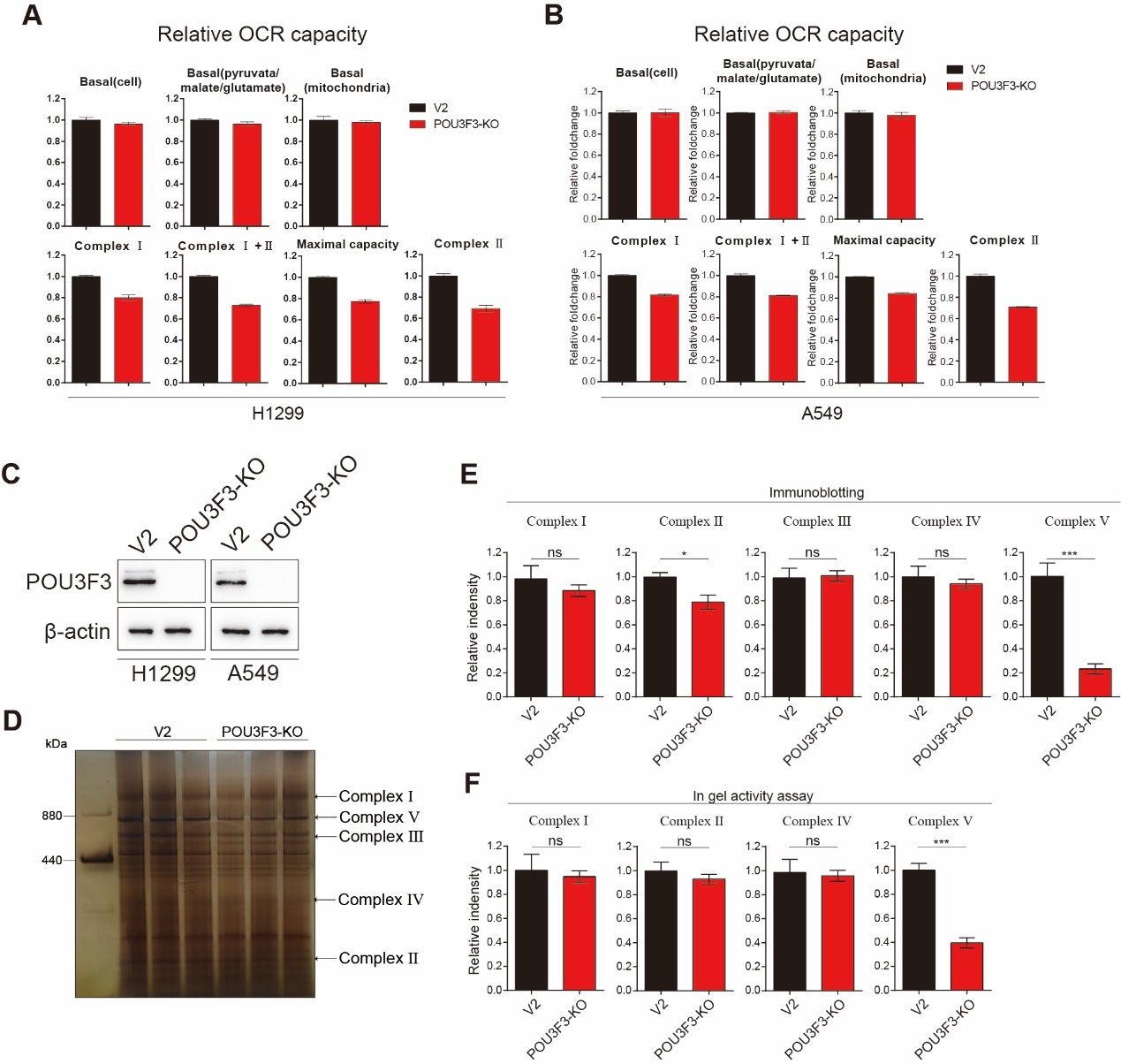


Figure S2. A and B. Statistical graph of effects of stably knockout of POU3F3 on mitochondrial respiration of H1299. C. Western blot confirmed the loading amount of H1299 and A549 cells measured by OCR and verified the knockout status of POU3F3. D. Analysis of subunits of the mitochondrial complexes from H1299 cell by BN-PAGE and subsequent silver staining. E and F. Relative density of immunoblotting bands (E) and in gel activity bands (F) of mitochondrial complexes. Results represent three independent experiments and are presented as mean ± SD (*p < 0.05, ⁎⁎p < 0.01, ⁎⁎⁎p < 0.001 vs. control group).


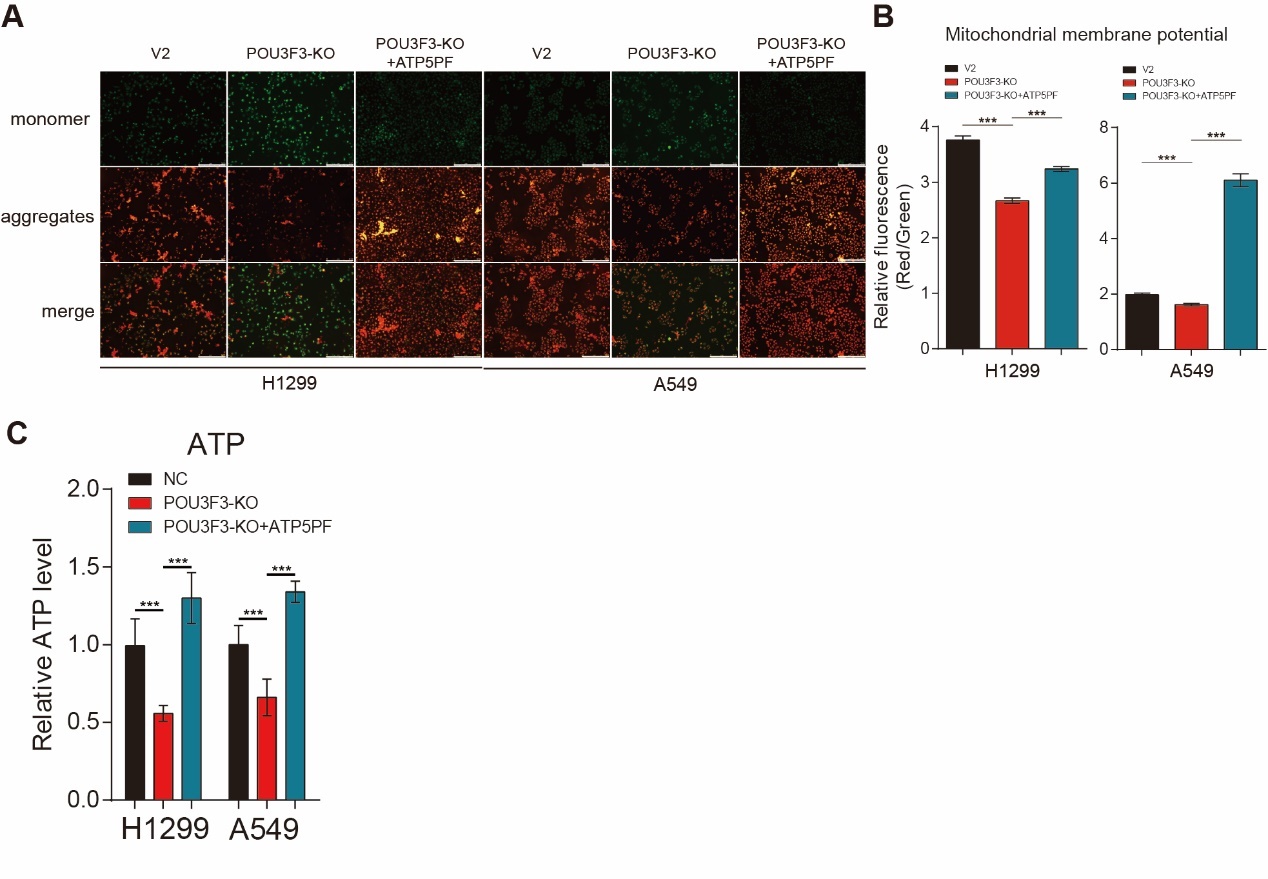


Figure S3. A and B. Inverted fluorescent microscope images (A) and Spectrophotometer (B) displayed the MMP in POU3F3 knockout cell and ATP5PF rescued H1299 and A549 cells. C. Rescue ATP5PF increased cellular ATP production in H1299 and A549 cells. Results represent three independent experiments and are presented as mean ± SD (*p < 0.05, ⁎⁎p < 0.01, ⁎⁎⁎p < 0.001 vs. control group).


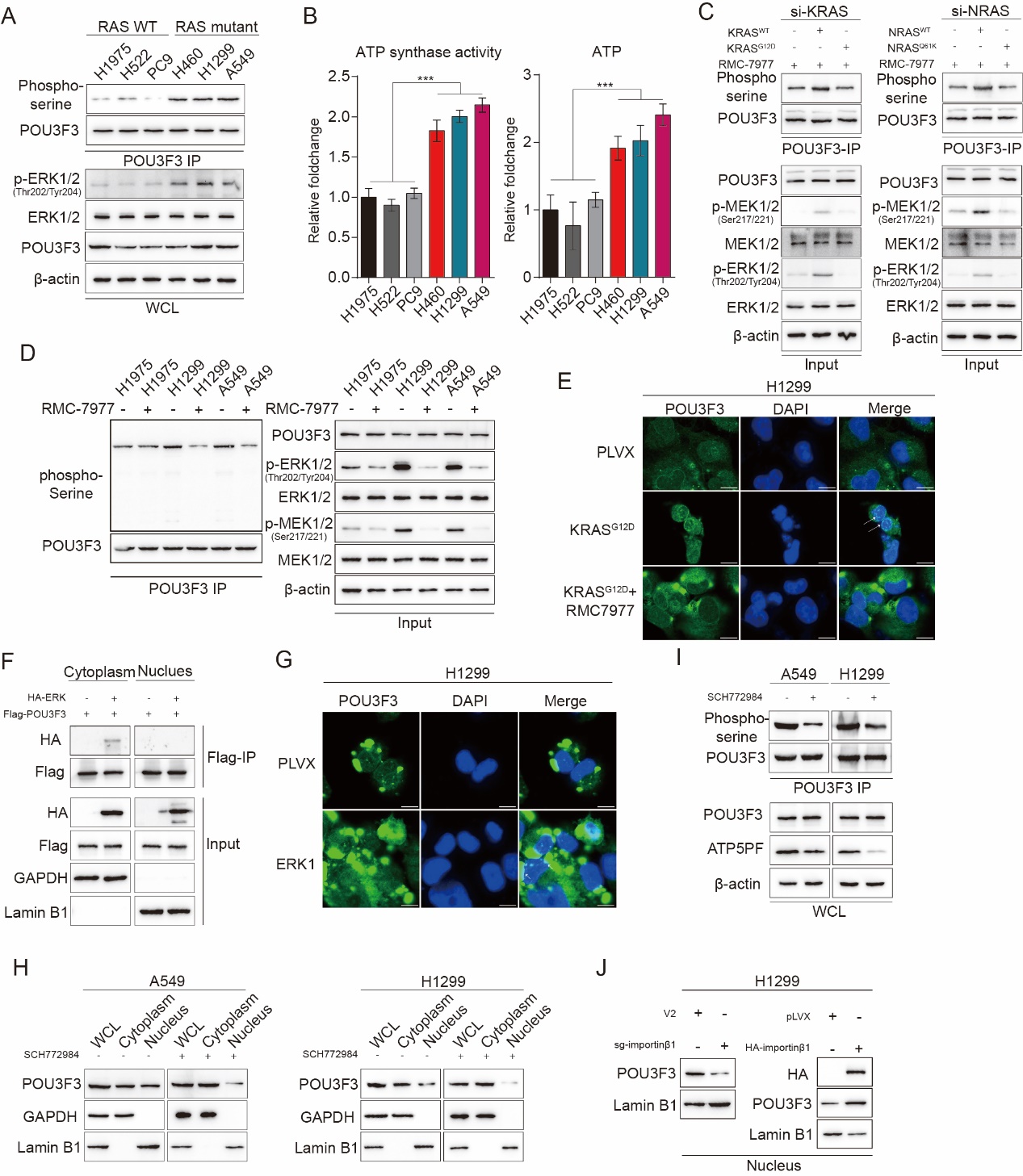


Figure S4. A The expression and phosphorylation level of POU3F3 in H1975, H522, PC9 and H460, H1299, A549 cells. B. The ATP synthase activity and cellular ATP production of H1975, H522, PC9 and H460, H1299, A549. Results represent three independent experiments and are presented as mean ± SD (*p < 0.05, ⁎⁎p < 0.01, ⁎⁎⁎p < 0.001 vs. control group). C. Western blot indicated that KRAS^G12D^ promoted phosphorylation of POU3F3, which RMC-7977 reversed this effect by specific inhibiting mutant RAS. D. RAS mutation cell line A549 and H1299 exhibited upregulated the phosphorylation of POU3F3 compared to RAS WT cell line H1975. E. Confocal microscope images displayed overexpression of KRAS^G12D^ translocated POU3F3 into nucleus. F. Western blot display that POU3F3 interact with ERK1 in cytoplasm. G. Confocal microscope images displayed overexpression of KRAS^G12D^ translocated POU3F3 into nucleus. H. Western blot exhibited that SCH772984 suppressed the nucleus location of POU3F3 and the expression of ATP5PF. I. Western blot exhibited that SCH772984 suppressed the phosphorylation of POU3F3. J. Western blot demonstrated that improtin β1 mediated POU3F3 translocation to nucleus.

**References**

[1] N. D. Machado, L. C. Heather, A. L. Harris, G. S. Higgins, *Br J Cancer* **2023**, *129* (6), 897, https://doi.org/10.1038/s41416-023-02394-9.

[2] a) C. Uslu, E. Kapan, A. Lyakhovich, *Cancer Lett* **2024**, *587*, 216705, https://doi.org/10.1016/j.canlet.2024.216705; b) Z. Zhao, Y. Mei, Z. Wang, W. He, *Cancers (Basel)* **2022**, *15* (1), https://doi.org/10.3390/cancers15010062.

[3] R. L. Siegel, K. D. Miller, N. S. Wagle, A. Jemal, *CA Cancer J Clin* **2023**, *73* (1), 17, https://doi.org/10.3322/caac.21763.

[4] Y. Zhang, S. Vaccarella, E. Morgan, M. Li, J. Etxeberria, E. Chokunonga, S. S. Manraj, B. Kamate, A. Omonisi, F. Bray, *Lancet Oncol* **2023**, *24* (11), 1206, https://doi.org/10.1016/s1470-2045(23)00444-8.

[5] a) T. B. Kratzer, P. Bandi, N. D. Freedman, R. A. Smith, W. D. Travis, A. Jemal, R. L. Siegel, *Cancer* **2024**, *130* (8), 1330, https://doi.org/10.1002/cncr.35128; b) Y. Li, B. Yan, S. He, *Biomed Pharmacother* **2023**, *169*, 115891, https://doi.org/10.1016/j.biopha.2023.115891.

[6] a) C. E. Wenner, M. A. Spirtes, S. Weinhouse, *Cancer Res* **1952**, *12* (1), 44; b) V. R. Fantin, J. St-Pierre, P. Leder, *Cancer Cell* **2006**, *9* (6), 425, https://doi.org/10.1016/j.ccr.2006.04.023.

[7] a) O. Warburg, *Science* **1956**, *123* (3191), 309, https://doi.org/10.1126/science.123.3191.309; b) J. Lu, M. Tan, Q. Cai, *Cancer Lett* **2015**, *356* (2 Pt A), 156, https://doi.org/10.1016/j.canlet.2014.04.001; c) W. X. Zong, J. D. Rabinowitz, E. White, *Mol Cell* **2016**, *61* (5), 667, https://doi.org/10.1016/j.molcel.2016.02.011.

[8] Z. Wu, M. Zuo, L. Zeng, K. Cui, B. Liu, C. Yan, L. Chen, J. Dong, F. Shangguan, W. Hu, H. He, B. Lu, Z. Song, *EMBO Rep* **2021**, *22* (1), e50827, https://doi.org/10.15252/embr.202050827.

[9] a) J. Greene, A. Segaran, S. Lord, *Semin Cancer Biol* **2022**, *86* (Pt 2), 851, https://doi.org/10.1016/j.semcancer.2022.02.002; b) L. X. Zampieri, C. Silva-Almeida, J. D. Rondeau, P. Sonveaux, *Int J Mol Sci* **2021**, *22* (6), https://doi.org/10.3390/ijms22063245.

[10] a) I. A. Prior, F. E. Hood, J. L. Hartley, *Cancer Res* **2020**, *80* (14), 2969, https://doi.org/10.1158/0008-5472.Can-19-3682; b) M. Malumbres, M. Barbacid, *Nat Rev Cancer* **2003**, *3* (6), 459, https://doi.org/10.1038/nrc1097.

[11] S. Guerrero, I. Casanova, L. Farré, A. Mazo, G. Capellà, R. Mangues, *Cancer Res* **2000**, *60* (23), 6750.

[12] A. A. Krygowska, E. Castellano, *Cold Spring Harb Perspect Med* **2018**, *8* (6), https://doi.org/10.1101/cshperspect.a031450.

[13] G. Maik-Rachline, A. Hacohen-Lev-Ran, R. Seger, *Int J Mol Sci* **2019**, *20* (5), https://doi.org/10.3390/ijms20051194.

[14] a) M. Deak, A. D. Clifton, L. M. Lucocq, D. R. Alessi, *Embo j* **1998**, *17* (15), 4426, https://doi.org/10.1093/emboj/17.15.4426; b) P. Gupta, R. Prywes, *J Biol Chem* **2002**, *277* (52), 50550, https://doi.org/10.1074/jbc.M209799200.

[15] G. Yin, J. Huang, J. Petela, H. Jiang, Y. Zhang, S. Gong, J. Wu, B. Liu, J. Shi, Y. Gao, *Signal Transduct Target Ther* **2023**, *8* (1), 212, https://doi.org/10.1038/s41392-023-01441-4.

[16] a) C. Cosse-Etchepare, I. Gervi, I. Buisson, L. Formery, M. Schubert, J. F. Riou, M. Umbhauer, R. Le Bouffant, *Int J Dev Biol* **2018**, *62* (4-5), 325, https://doi.org/10.1387/ijdb.170260RL; b) L. Snijders Blok, T. Kleefstra, H. Venselaar, S. Maas, H. Y. Kroes, A. M. A. Lachmeijer, K. L. I. van Gassen, H. V. Firth, S. Tomkins, S. Bodek, K. Õunap, M. H. Wojcik, C. Cunniff, K. Bergstrom, Z. Powis, S. Tang, D. N. Shinde, C. Au, A. D. Iglesias, K. Izumi, J. Leonard, A. Abou Tayoun, S. W. Baker, M. Tartaglia, M. Niceta, M. L. Dentici, N. Okamoto, N. Miyake, N. Matsumoto, A. Vitobello, L. Faivre, C. Philippe, C. Gilissen, L. Wiel, R. Pfundt, P. Deriziotis, H. G. Brunner, S. E. Fisher, *Am J Hum Genet* **2019**, *105* (2), 403, https://doi.org/10.1016/j.ajhg.2019.06.007.

[17] a) J. Ishii, H. Sato, M. Sakaeda, Y. Shishido-Hara, C. Hiramatsu, H. Kamma, H. Shimoyamada, M. Fujiwara, T. Endo, I. Aoki, T. Yazawa, *Pathol Int* **2013**, *63* (3), 158, https://doi.org/10.1111/pin.12042; b) M. Sakaeda, H. Sato, J. Ishii, C. Miyata, H. Kamma, Y. Shishido-Hara, H. Shimoyamada, M. Fujiwara, T. Endo, R. Tanaka, H. Kondo, T. Goya, I. Aoki, T. Yazawa, *Lab Invest* **2013**, *93* (4), 408, https://doi.org/10.1038/labinvest.2013.2.

[18] a) I. Wittig, H. P. Braun, H. Schägger, *Nat Protoc* **2006**, *1* (1), 418, https://doi.org/10.1038/nprot.2006.62; b) G. Beutner, G. A. Porter, Jr., *J Vis Exp* **2017**, (124), https://doi.org/10.3791/55738.

[19] T. J. Kilbaugh, M. Karlsson, M. Byro, A. Bebee, J. Ralston, S. Sullivan, A. C. Duhaime, M. J. Hansson, E. Elmér, S. S. Margulies, *Exp Neurol* **2015**, *271*, 136, https://doi.org/10.1016/j.expneurol.2015.05.009.

[20] A. Subramanian, P. Tamayo, V. K. Mootha, S. Mukherjee, B. L. Ebert, M. A. Gillette, A. Paulovich, S. L. Pomeroy, T. R. Golub, E. S. Lander, J. P. Mesirov, *Proc Natl Acad Sci U S A* **2005**, *102* (43), 15545, https://doi.org/10.1073/pnas.0506580102.

[21] E. Cerami, J. Gao, U. Dogrusoz, B. E. Gross, S. O. Sumer, B. A. Aksoy, A. Jacobsen, C. J. Byrne, M. L. Heuer, E. Larsson, Y. Antipin, B. Reva, A. P. Goldberg, C. Sander, N. Schultz, *Cancer Discov* **2012**, *2* (5), 401, https://doi.org/10.1158/2159-8290.Cd-12-0095.

[22] B. Győrffy, *Innovation (Camb)* **2024**, *5* (3), 100625, https://doi.org/10.1016/j.xinn.2024.100625.

[23] a) Y. Liu, Y. Zhuang, X. Fu, C. Li, *Discov Oncol* **2021**, *12* (1), 21, https://doi.org/10.1007/s12672-021-00414-9; b) K. Wu, Q. Wang, Y. L. Liu, Z. Xiang, Q. Q. Wang, L. Yin, S. L. Liu, *Front Oncol* **2021**, *11*, 643613, https://doi.org/10.3389/fonc.2021.643613; c) M. Shi, M. Sun, C. Wang, Y. Shen, Y. Wang, S. Yan, *Curr Gene Ther* **2022**, *22* (5), 427, https://doi.org/10.2174/1566523222666220309150722.

[24] Q. Zeng, Y. Dai, C. Duan, R. Zeng, Q. Zeng, C. Wei, *BMC Pulm Med* **2020**, *20* (1), 185, https://doi.org/10.1186/s12890-020-01218-3.

[25] W. Li, J. Zheng, J. Deng, Y. You, H. Wu, N. Li, J. Lu, Y. Zhou, *Gastroenterology* **2014**, *146* (7), 1714, https://doi.org/10.1053/j.gastro.2014.03.002.

[26] J. Q. Xu, Y. L. Fu, J. Zhang, K. Y. Zhang, J. Ma, J. Y. Tang, Z. W. Zhang, Z. Y. Zhou, *Front Pharmacol* **2022**, *13*, 1037341, https://doi.org/10.3389/fphar.2022.1037341.

[27] a) T. Wang, F. Sun, C. Li, P. Nan, Y. Song, X. Wan, H. Mo, J. Wang, Y. Zhou, Y. Guo, A. E. Helali, D. Xu, Q. Zhan, F. Ma, H. Qian, *Adv Sci (Weinh)* **2023**, *10* (25), e2300756, https://doi.org/10.1002/advs.202300756; b) L. He, J. Lin, S. Lu, H. Li, J. Chen, X. Wu, Q. Yan, H. Liu, H. Li, Y. Shi, *Adv Sci (Weinh)* **2024**, e2403093, https://doi.org/10.1002/advs.202403093.

[28] Y. Lai, Y. Zhang, S. Zhou, J. Xu, Z. Du, Z. Feng, L. Yu, Z. Zhao, W. Wang, Y. Tang, X. Yang, L. W. Guddat, F. Liu, Y. Gao, Z. Rao, H. Gong, *Mol Cell* **2023**, *83* (12), 2137, https://doi.org/10.1016/j.molcel.2023.04.029.

[29] M. Reck, D. P. Carbone, M. Garassino, F. Barlesi, *Ann Oncol* **2021**, *32* (9), 1101, https://doi.org/10.1016/j.annonc.2021.06.001.

[30] M. Holderfield, B. J. Lee, J. Jiang, A. Tomlinson, K. J. Seamon, A. Mira, E. Patrucco, G. Goodhart, J. Dilly, Y. Gindin, N. Dinglasan, Y. Wang, L. P. Lai, S. Cai, L. Jiang, N. Nasholm, N. Shifrin, C. Blaj, H. Shah, J. W. Evans, N. Montazer, O. Lai, J. Shi, E. Ahler, E. Quintana, S. Chang, A. Salvador, A. Marquez, J. Cregg, Y. Liu, A. Milin, A. Chen, T. B. Ziv, D. Parsons, J. E. Knox, J. E. Klomp, J. Roth, M. Rees, M. Ronan, A. Cuevas-Navarro, F. Hu, P. Lito, D. Santamaria, A. J. Aguirre, A. M. Waters, C. J. Der, C. Ambrogio, Z. Wang, A. L. Gill, E. S. Koltun, J. A. M. Smith, D. Wildes, M. Singh, *Nature* **2024**, *629* (8013), 919, https://doi.org/10.1038/s41586-024-07205-6.

[31] a) A. S. Futran, A. J. Link, R. Seger, S. Y. Shvartsman, *Curr Biol* **2013**, *23* (21), R972, https://doi.org/10.1016/j.cub.2013.09.033; b) Z. T. Kelleher, C. Wang, M. T. Forrester, M. W. Foster, H. E. Marshall, *J Biol Chem* **2019**, *294* (36), 13336, https://doi.org/10.1074/jbc.RA119.007733.

[32] M. D. Schonemann, A. K. Ryan, L. Erkman, R. J. McEvilly, J. Bermingham, M. G. Rosenfeld, *Adv Exp Med Biol* **1998**, *449*, 39, https://doi.org/10.1007/978-1-4615-4871-3_4.

[33] C. Fionda, D. Di Bona, A. Kosta, H. Stabile, A. Santoni, M. Cippitelli, *Cancers (Basel)* **2019**, *11* (6), https://doi.org/10.3390/cancers11060810.

[34] Y. H. Huang, O. Klingbeil, X. Y. He, X. S. Wu, G. Arun, B. Lu, T. D. D. Somerville, J. P. Milazzo, J. E. Wilkinson, O. E. Demerdash, D. L. Spector, M. Egeblad, J. Shi, C. R. Vakoc, *Genes Dev* **2018**, *32* (13-14), 915, https://doi.org/10.1101/gad.314815.118.

[35] J. L. Simmons, C. J. Pierce, F. Al-Ejeh, G. M. Boyle, *Sci Rep* **2017**, *7* (1), 10909, https://doi.org/10.1038/s41598-017-11366-y.

[36] a) M. L. Suvà, E. Rheinbay, S. M. Gillespie, A. P. Patel, H. Wakimoto, S. D. Rabkin, N. Riggi, A. S. Chi, D. P. Cahill, B. V. Nahed, W. T. Curry, R. L. Martuza, M. N. Rivera, N. Rossetti, S. Kasif, S. Beik, S. Kadri, I. Tirosh, I. Wortman, A. K. Shalek, O. Rozenblatt-Rosen, A. Regev, D. N. Louis, B. E. Bernstein, *Cell* **2014**, *157* (3), 580, https://doi.org/10.1016/j.cell.2014.02.030; b) A. Bogeas, G. Morvan-Dubois, E. A. El-Habr, F. X. Lejeune, M. Defrance, A. Narayanan, K. Kuranda, F. Burel-Vandenbos, S. Sayd, V. Delaunay, L. G. Dubois, H. Parrinello, S. Rialle, S. Fabrega, A. Idbaih, J. Haiech, I. Bièche, T. Virolle, M. Goodhardt, H. Chneiweiss, M. P. Junier, *Acta Neuropathol* **2018**, *135* (2), 267, https://doi.org/10.1007/s00401-017-1783-x.

[37] a) D. J. Sharpe, K. S. Orr, M. Moran, S. J. White, S. McQuaid, T. R. Lappin, A. Thompson, J. A. James, *Oncotarget* **2014**, *5* (18), 8803, https://doi.org/10.18632/oncotarget.2492; b) H. Y. Zhu, G. Y. Cao, S. P. Wang, Y. Chen, G. D. Liu, Y. J. Gao, J. P. Hu, *Am J Cancer Res* **2017**, *7* (8), 1665.

[38] H. Lopez-Bertoni, I. S. Kotchetkov, N. Mihelson, B. Lal, Y. Rui, H. Ames, M. Lugo-Fagundo, H. Guerrero-Cazares, A. Quiñones-Hinojosa, J. J. Green, J. Laterra, *Cancer Res* **2020**, *80* (8), 1644, https://doi.org/10.1158/0008-5472.Can-19-1624.

[39] R. M. Pascale, D. F. Calvisi, M. M. Simile, C. F. Feo, F. Feo, *Cancers (Basel)* **2020**, *12* (10), https://doi.org/10.3390/cancers12102819.

[40] P. S. Ward, C. B. Thompson, *Cancer Cell* **2012**, *21* (3), 297, https://doi.org/10.1016/j.ccr.2012.02.014.

[41] a) Y. Lissanu Deribe, Y. Sun, C. Terranova, F. Khan, J. Martinez-Ledesma, J. Gay, G. Gao, R. A. Mullinax, T. Khor, N. Feng, Y. H. Lin, C. C. Wu, C. Reyes, Q. Peng, F. Robinson, A. Inoue, V. Kochat, C. G. Liu, J. M. Asara, C. Moran, F. Muller, J. Wang, B. Fang, V. Papadimitrakopoulou, Wistuba, II, K. Rai, J. Marszalek, P. A. Futreal, *Nat Med* **2018**, *24* (7), 1047, https://doi.org/10.1038/s41591-018-0019-5; b) X. L. Zu, M. Guppy, *Biochem Biophys Res Commun* **2004**, *313* (3), 459, https://doi.org/10.1016/j.bbrc.2003.11.136; c) A. Viale, D. Corti, G. F. Draetta, *Cancer Res* **2015**, *75* (18), 3685, https://doi.org/10.1158/0008-5472.Can-15-0491; d) T. M. Ashton, W. G. McKenna, L. A. Kunz-Schughart, G. S. Higgins, *Clin Cancer Res* **2018**, *24* (11), 2482, https://doi.org/10.1158/1078-0432.Ccr-17-3070.

[42] a) C. R. Lindsay, M. C. Garassino, E. Nadal, K. Öhrling, M. Scheffler, J. Mazières, *Lung Cancer* **2021**, *160*, 152, https://doi.org/10.1016/j.lungcan.2021.07.005; b) T. K. H. Lim, F. Skoulidis, K. M. Kerr, M. J. Ahn, J. R. Kapp, F. A. Soares, Y. Yatabe, *Lung Cancer* **2023**, *184*, 107293, https://doi.org/10.1016/j.lungcan.2023.107293.

[43] a) G. J. Riely, M. L. Johnson, C. Medina, N. A. Rizvi, V. A. Miller, M. G. Kris, M. C. Pietanza, C. G. Azzoli, L. M. Krug, W. Pao, M. S. Ginsberg, *J Thorac Oncol* **2011**, *6* (8), 1435, https://doi.org/10.1097/JTO.0b013e318223c099; b) P. A. Jänne, M. M. van den Heuvel, F. Barlesi, M. Cobo, J. Mazieres, L. Crinò, S. Orlov, F. Blackhall, J. Wolf, P. Garrido, A. Poltoratskiy, G. Mariani, D. Ghiorghiu, E. Kilgour, P. Smith, A. Kohlmann, D. J. Carlile, D. Lawrence, K. Bowen, J. Vansteenkiste, *Jama* **2017**, *317* (18), 1844, https://doi.org/10.1001/jama.2017.3438.

[44] M. B. Ryan, R. B. Corcoran, *Nat Rev Clin Oncol* **2018**, *15* (11), 709, https://doi.org/10.1038/s41571-018-0105-0.

[45] T. Suzuki, T. Kishikawa, T. Sato, N. Takeda, Y. Sugiura, T. Seimiya, K. Sekiba, M. Ohno, T. Iwata, R. Ishibashi, M. Otsuka, K. Koike, *Cancer Gene Ther* **2022**, *29* (5), 505, https://doi.org/10.1038/s41417-021-00326-4.

[46] S. S. Hu, Y. Han, T. Y. Tan, H. Chen, J. W. Gao, L. Wang, M. H. Yang, L. Zhao, Y. Q. Wang, Y. Q. Ding, S. Wang, *JCI Insight* **2023**, *8* (21), https://doi.org/10.1172/jci.insight.167874.

[47] J. Feng, Z. Lian, X. Xia, Y. Lu, K. Hu, Y. Zhang, Y. Liu, L. Hu, K. Yuan, Z. Sun, X. Pang, *Acta Pharm Sin B* **2023**, *13* (3), 1145, https://doi.org/10.1016/j.apsb.2022.10.023.

[48] a) L. Schöckel, A. Glasauer, F. Basit, K. Bitschar, H. Truong, G. Erdmann, C. Algire, A. Hägebarth, P. H. Willems, C. Kopitz, W. J. Koopman, M. Héroult, *Cancer Metab* **2015**, *3*, 11, https://doi.org/10.1186/s40170-015-0138-0; b) L. Helbig, L. Koi, K. Brüchner, K. Gurtner, H. Hess-Stumpp, K. Unterschemmann, M. Baumann, D. Zips, A. Yaromina, *Radiat Oncol* **2014**, *9*, 207, https://doi.org/10.1186/1748-717x-9-207; c) R. Ju, K. Fei, S. Li, C. Chen, L. Zhu, J. Li, D. Zhang, L. Guo, C. Ye, *J Pharmacol Exp Ther* **2018**, *367* (1), 20, https://doi.org/10.1124/jpet.118.249326; d) J. R. Diamond, B. Goff, M. D. Forster, J. C. Bendell, C. D. Britten, M. S. Gordon, H. Gabra, D. M. Waterhouse, M. Poole, D. Ross Camidge, E. Hamilton, K. M. Moore, *Invest New Drugs* **2017**, *35* (5), 627, https://doi.org/10.1007/s10637-017-0444-1.
